# Global, regional, and national burden of cardiovascular disease due to dietary risks, 1990–2021

**DOI:** 10.3389/fnut.2025.1623855

**Published:** 2025-10-01

**Authors:** Yunfeng Yu, Danni Tan, Xinyu Yang, Jingyi Wu, Gang Hu, Weixiong Jian, Liping Wang

**Affiliations:** ^1^School of Traditional Chinese Medicine, Hunan University of Chinese Medicine, Changsha, Hunan, China; ^2^School of Traditional Chinese Medicine, Hunan University of Medicine, Huaihua, Hunan, China; ^3^The Third School of Clinical Medicine, Zhejiang Chinese Medical University, Hangzhou, Zhejiang, China

**Keywords:** dietary risk, hypertensive heart disease, stroke, Global Burden of Disease, ischemic heart disease, cardiovascular disease

## Abstract

**Objective:**

This study investigated the burden and temporal trends of cardiovascular disease due to dietary risk (CVD-DR) from 1990 to 2021.

**Methods:**

This study used the Global Burden of Disease (GBD) 2021 database to calculate the estimated deaths, age-standardized mortality rate (ASMR), disability-adjusted life years (DALYs), age-standardized DALY rate (ASDR), and annual percentage change (EAPC) from CVD-DR from 1990 to 2021. We then compared the ASDRs and ASMRs of CVD-DR according to region, socio-demographic index (SDI), sex, and age group. Finally, we evaluated the burdens of cardiovascular disease (CVD) induced by 13 different dietary risks.

**Results:**

From 1990 to 2021, the global DALYs and deaths from CVD-DR increased by 36.5% and 44.8%, respectively, whereas the ASDR and ASMR decreased to 62.9% and 61.4% of the baseline values, respectively. In 2021, 5,833,851 deaths and 134,179,728 DALYs from CVD-DR were reported, with an ASMR of 69.81 per 100,000 population and an ASDR of 1,563.86 per 100,000 population. Hypertensive heart disease (HHD), stroke, and ischemic heart disease (IHD) due to dietary risks were the primary components. Correlation analyses indicated that ASMR and ASDR and their EAPCs were negatively correlated with SDI (ρ < 0, *P* < 0.05). Among the 13 dietary risk factors, a diet high in sodium, low in fruits, and low in whole grains was the main dietary risk factor for CVD.

**Conclusion:**

Over the last 31 years, the overall global burden of CVD-DR has increased, whereas the age-standardized burden has decreased, with the burdens mainly originating from IHD, stroke, and HHD. Lower-SDI regions face a higher and faster-growing burden of CVD-DR. Diets high in sodium and low in fruits and whole grains were the leading dietary contributors to this burden.

## 1 Introduction

Cardiovascular disease (CVD) encompasses a range of vascular and cardiac diseases characterized by vascular stenosis or occlusion and includes stroke, arrhythmia, ischemic heart disease (IHD), and peripheral arterial disease (1). CVD is the primary cause of death worldwide and a major challenge to global health (2). An epidemiological study showed that approximately 17.9 million people worldwide died of CVD in 2019, accounting for 32% of global deaths (3). Stroke and IHD are the primary causes of mortality in patients with CVD (3). Approximately 34% of global CVD deaths are premature, although the proportions of such deaths vary worldwide, including 22% in Europe and 35% in Asia (4). Moreover, the mortality rate of CVD is associated with economic level, as the World Health Organization (WHO) reported that over 75% of CVD-related deaths occur in low- and middle-income countries (3). CVD not only affects patient functional tasks and quality of life but also imposes tremendous economic burdens on patients’ families and society (5). In 2010, the direct and indirect medical costs related to CVD in the United States totaled 273 billion and 172 billion US dollars, respectively (6), of which stroke and coronary heart disease (CHD) accounted for 15.6 billion and 50.8 billion in direct healthcare costs (7). Additionally, in 2021, the health expenditure on CVD in the European Union reached 282 billion US dollars, representing 11% of the total health spending (8). Taken together, these findings highlight CVD as a persistent global public health challenge and a leading threat to human health.

The high-risk factors for CVD, including obesity, hypertension, and dyslipidemia (9), are all strongly associated with unhealthy diets (3). A cohort study in France showed that ultra-processed food intake increased CVD risk by 12% (hazard ratio [HR] 1.12, 95% confidence interval [CI] 1.05–1.20), CHD by 13% (HR 1.13, 95% CI 1.02–1.24), and cerebrovascular disease by 11% (HR 1.11, 95% CI 1.01–1.21) (10). A subsequent meta-analysis indicated that a total dietary fiber intake of 7 g/day reduced CVD risk by 9% (risk ratio [RR] 0.91, 95% CI 0.88–0.94) and CHD by 9% (RR 0.91, 95% CI 0.87–0.94) (11). Conversely, a diet lacking dietary fiber increases the risks of CVD and CHD (11). Therefore, dietary strategies for CVD prevention recommend a dietary fiber intake of 25–40 g/day, a vegetable and fruit intake of 200 g/day, and a salt intake of <5 g/day (9). Additionally, some studies have recommended that patients with obesity and hypercholesterolemia limit saturated fatty acid intake to 10% and 7% of the total caloric intake, respectively, and increase the intake of monounsaturated and polyunsaturated fatty acids (9). Together, these findings highlight the dual role of dietary patterns in influencing CVD risk. Therefore, focusing on the main dietary risk factors for CVD and their prevalence trends in different regions is important for facilitating the development of targeted dietary management policies for CVD. However, the global and regional prevalence and mortality of CVD attributable to dietary risks (CVD-DR) remain unclear, highlighting the need for a detailed assessment of its burden and contributing factors.

The Global Burden of Disease (GBD) is a large-scale international research initiative that quantifies the health impacts of diseases and risk factors worldwide (12). Using data from 204 countries and territories, it provides a comprehensive resource for investigating the distribution and trends of specific diseases (12). Using the GBD 2021 data, we developed a multidimensional analytical framework to evaluate CVD-DR across 204 countries and territories over 31 years (1990–2021). Furthermore, we examined the impact of regional development level on disease burden through association modeling and assessed differences across sex and age groups, with the goal of informing policy-making.

## 2 Materials and methods

### 2.1 Study design

Using the GBD 2021 data, we analyzed the number of deaths, disability-adjusted life years (DALYs), age-standardized DALY rate (ASDR), age-standardized mortality rate (ASMR), and estimated annual percentage change (EAPC) of CVD-DR worldwide. We further examined the CVD-DR burden across regions, sociodemographic indices, sexes, and ages. Because these data are publicly available, no additional ethical approval was required for this study.

### 2.2 Data sources

The dataset for this study was obtained from the GBD 2021 data (13). CVD was coded as I51.6 according to the International Classification of Diseases, Tenth Revision (ICD-10). We obtained CVD data from the Global Health Data Exchange^1^, including six subtypes of CVD: IHD, stroke, lower extremity peripheral artery disease (LEPAD), aortic aneurysm (AA), atrial fibrillation and flutter (AFF), and hypertensive heart disease (HHD). The ICD-10 code for IHD is I20-I25, representing a condition affecting the coronary arteries, typically due to atherosclerosis, which results in angina, myocardial infarction, or ischemic cardiomyopathy. Stroke is coded as I64 and is defined by the rapid onset of focal disturbance of cerebral function persisting for >24 h or resulting in death. HHD, coded as I11, refers to a range of heart conditions induced by long-term high blood pressure that can lead to heart failure, with either preserved or reduced systolic function of the left ventricle. AFF, coded as I48, is an arrhythmia resulting from electrical conduction problems affecting the heart atria. AA is coded as I71.9 and is characterized by abnormal enlargement and weakening of the abdominal or thoracic aorta due to inflammation, high blood pressure, changes in cellular or extracellular structure, or atherosclerosis, resulting in blood vessel tear or rupture. LEPAD is coded as I73.9 and is characterized by an ankle-brachial index of <0.90.

We collected data on 15 dietary risk factors, stratified by sex and age groups for individuals aged ≥25 years from the GBD, and 13 specific dietary risks associated with CVD from the GHDX (accessed April 5, 2025; the definitions are presented in Supplementary Table 1). This included diets high in trans fatty acids, sugar-sweetened beverages, processed meat, sodium, and red meat, as well as diets low in vegetables, fruits, whole grains, seafood omega-3 fatty acids, nuts and seeds, fiber, legumes, and polyunsaturated fatty acids.

The GBD uses a counterfactual framework that defines a theoretical minimum risk exposure level (TMREL) for each risk factor independently, without assuming changes in co-occurring exposures. To address overlapping effects and mediated pathways among multiple risk factors, the GBD methodology incorporates a mediation matrix. This approach prevents overestimation of the combined burden attributable to multiple risks and ensures internal consistency in calculating population-attributable fractions (PAFs). Therefore, the CVD-DR burden estimated in this study reflects the impact of dietary factors itself, without the confounding influence of other risk factors.

### 2.3 Statistical analysis

All statistical analyses and visualizations were performed using R software (version 4.4.1). We assessed the global burden and temporal trends of CVD-DR according to the number of deaths, DALYs, ASMRs, ASDRs, and EAPCs. The deaths were defined as the number of individuals who died of CVD-DR in a given year. DALY was calculated as the sum of years of life lost (YLL) due to premature mortality and years lived with disability (YLD) due to disease, which reflected the overall disease burden, as follows: DALY = YLL + YLD. We determined the age-standardized rate (ASR) as the weighted average of age-specific rates, where the weights were the proportions of each age group in a standard population, including the ASMR and ASDR. ASMR refers to the age-standardized rate per 100,000 deaths, and ASDR as the age-standardized DALY rate per 100,000 population. We calculated the ASR using the following formula: $A\,S\,R\,\sum_{i\,1}^{A}a_{i}\,w_{i}/\sum_{i\,1}^{A}w_{i}\,100,000$, where *a*_*i*_ represents the *i-*th age group and *w*_*i*_ is the number (or proportion) of the population in the same age group in the GBD world standard population. All estimates are presented with 95% uncertainty intervals (UIs) derived from the GBD 2021 study framework. These UIs represent Bayesian credible intervals calculated from 1,000 posterior draws that incorporate uncertainties from incomplete data, model assumptions, and measurement errors. The 2.5th and 97.5th percentiles of the ordered draws are considered the lower and upper bounds, respectively, indicating a 95% probability that the true value lies within this range. The EAPC quantifies the average annual percentage change in ASMR or ASDR over a specified period. We assessed these temporal trends using a linear regression model, based on the equation Y = α+βX+ε, where Y is the natural logarithm of the ASR, X denotes the calendar year, and ε is the error term. We calculated EAPC using the formula EAPC = 100 × (exp (β) - 1), which was presented as numerical values and 95% confidence intervals (CIs). EAPC >0 indicated an increasing trend, whereas EAPC <0 indicated a decreasing trend.

We conducted subgroup analyses to comprehensively evaluate heterogeneity in CVD-DR burden. First, we analyzed 21 GBD regions and 204 countries or territories to capture geographical variations. Second, we stratified all countries or territories into five sociodemographic index (SDI) categories (low, low-middle, middle, high-middle, and high) according to the GBD classification. The SDI is a composite measure based on lag-distributed income per capita, average educational attainment in individuals aged ≥15 years, and total fertility rate in women aged <25 years. We assessed the correlation between SDI and disease burden (ASMR, ASDR, and their EAPCs) using the Spearman correlation coefficient (ρ), with |ρ| values close to 1 indicating a strong monotonic relationship and *p* < 0.05 considered statistically significant. Third, we conducted sex-specific analyses to compare burdens between males and females. Fourth, we grouped individuals aged ≥25 years into 15 consecutive 5-year intervals to evaluate age-related patterns in the ASMR and ASDR. Finally, we quantified the contributions of the 13 dietary risk factors to the burden of CVD and its major subtypes, using the GBD dietary risk definitions to highlight which dietary components most strongly drive disease burden.

## 3 Results

### 3.1 Global, regional, and national burdens

#### 3.1.1 CVD-DR

From 1990 to 2021, the global number of deaths from CVD-DR increased from 4,028,354 (95% UI 1,114,891–5,748,940) to 5,833,851 (95% UI 1,357,129–8,661,541), whereas ASMR decreased from 113.61 (95% UI 31.19–164.63) per 100,000 population to 69.81 (95% UI 16.19–104.09), with an EAPC of −1.69 (95% CI −1.74 to −1.64). Similarly, the global DALYs of CVD-DR increased from 98,331,777 (95% UI 27,092,345–136,036,264) to 134,179,728 (95% UI 32,581,870–192,859,591), whereas ASDR decreased from 2,487.47 (95% UI 675.52–3,480.84) per 100,000 population to 1,563.86 (95% UI 378.95–2,246.75), with an EAPC of −1.65 (95% CI −1.71 to −1.59). This indicated that although the absolute number of deaths rose, likely due to population growth and aging, the mortality risk for a standardized population declined, which may reflect progress in prevention, early detection, and treatment of CVD-DR. Southern Sub-Saharan Africa showed the fastest ASDR and ASMR growth during the study period, with EAPCs of 0.06 (95% CI −0.35 to 0.48) and 0.28 (95% CI −0.13 to 0.69), respectively. These increases point to a worsening health burden in this region, which may be related to persistent exposure to risk factors and limited medical resources. In 2021, Central Asia demonstrated the highest ASDR and ASMR, at 3,199.1 (95% UI 308.38–4,689.7) and 163.72 (95% UI 17.45–244.87) per 100,000 population, respectively. This reflects substantial health burdens in terms of mortality and years of life lost, potentially associated with high rates of hyper- tension, poor diet, tobacco use, and inadequate healthcare infrastructure. Tables 1, 2 present the global and regional burden and trends of CVD-DR.

**TABLE 1:** Deaths and ASMRs for CVD-DR and its temporal trends from 1990 to 2021.

Location	Death cases (95% UI)		ASMR (95% UI)		1990–2021
	1990	2021	1990	2021	EAPCs (95% CI)
**Global**	4,028,354 (1,114,891–5,748,940)	5,833,851 (1,357,129–8,661,541)	113.61 (31.19–164.63)	69.81 (16.19–104.09)	−1.69 (−1.74 to −1.64)
**SDI**					
High SDI	916,243 (171,383–1,390,206)	743,801 (226,697–1,125,742)	83.61 (15.48–126.75)	32.12 (9.77–47.84)	−3.19 (−3.28 to −3.1)
High-middle SDI	1,182,194 (305,581–1,693,233)	1,467,060 (364,918–2,200,628)	136.06 (32.98–198.86)	75.95 (18.69–114)	−2.19 (−2.41 to −1.97)
Middle SDI	1,050,404 (451,712–1,447,475)	1,921,191 (623,921–2,860,836)	120.94 (52.04–168.98)	78.74 (25.8–118.5)	−1.39 (−1.44 to −1.35)
Low-middle SDI	642,520 (135,999–927,133)	1,270,803 (219,491–1,888,872)	116.1 (26.92–168.71)	95.95 (18.16–143.76)	−0.57 (−0.62 to −0.51)
Low SDI	230,551 (63,053–329,760)	424,682 (119,799–612,234)	116.45 (35.01–168.73)	96.81 (30.01–139.76)	−0.61 (−0.7 to −0.52)
**Central Europe, Eastern Europe, and Central Asia**					
Central Asia	91,245 (16,504–129,412)	111,395 (11,167–164,463)	216.78 (40.58–311.31)	163.72 (17.45–244.87)	−1.39 (−1.71 to −1.07)
Central Europe	276,665 (99,985–384,736)	235,074 (92,237–339,647)	203.9 (73.89–284.63)	99.86 (38.93–143.91)	−2.59 (−2.69 to −2.5)
Eastern Europe	491,619 (47,186–743,582)	469,258 (8,066–756,623)	195.25 (16.59–298.33)	132.36 (2.67–212.34)	−1.91 (−2.41 to −1.4)
**High income region**					
Australasia	17,796 (−2,857–30,066)	12,266 (–802–20,542)	78.42 (–12.48–132.54)	20.38 (–1.41–33.69)	−4.57 (−4.7 to −4.45)
High-income Asia Pacific	90,796 (36,536–137,671)	94,540 (36,523–150,036)	50.55 (20.02–76.97)	15.99 (6.29–24.8)	−3.72 (−3.9 to −3.54)
High-income North America	289,103 (68,587–444,826)	259,054 (105,986–372,583)	81.03 (19.69–123.56)	38.13 (16.35–54.07)	−2.53 (−2.64 to −2.41)
Southern Latin America	43,587 (12,673–60,664)	35,149 (13,299–49,618)	102.45 (29.6–143.26)	38.92 (14.72–54.81)	−2.82 (−2.98 to −2.65)
Western Europe	439,501 (34,193–691,941)	293,096 (71,670–451,041)	74.98 (6.08–116.98)	25.67 (6.06–38.99)	−3.54 (−3.63 to −3.45)
**Latin America and Caribbean**					
Andean Latin America	12,097 (3,756–17,496)	19,674 (5,517–30,550)	64.66 (20.46–93.89)	34.53 (9.87–53.69)	−2.15 (−2.38 to −1.92)
Caribbean	24,400 (4,733–37,309)	32,704 (10,522–49,609)	101.55 (20.77–155.88)	60.04 (19.36–91.02)	−1.73 (−1.93 to −1.54)
Central Latin America	53,873 (13,864–78,885)	122,176 (22,863–186,856)	74.48 (19.1–110.2)	50.81 (9.47–78.13)	−1.44 (−1.68 to −1.2)
Tropical Latin America	70,799 (36,798–98,853)	88,211 (46,136–124,930)	86.79 (43.4–123.49)	35.08 (18.35–49.87)	−2.86 (−2.97 to −2.75)
**North Africa and Middle East**					
North Africa and Middle East	240,956 (24,531–359,090)	404,374 (27,418–653,728)	164.94 (19.52–250.9)	101.67 (8.74–164.31)	−1.67 (−1.73 to −1.61)
**South Asia**					
South Asia	572,731 (80,424–844,770)	1,351,733 (186,686–2,025,289)	107.07 (17.58–160.26)	98.54 (15.13–148.72)	−0.18 (−0.25 to −0.11)
**Southeast Asia, East Asia, and Oceania**					
East Asia	852,663 (484,606–1,181,876)	1,494,192 (617,767–2,305,249)	126.79 (69.05–178.77)	76.89 (30.43–119.06)	−1.49 (−1.6 to −1.37)
Oceania	3,660 (1,158–5,444)	8,163 (1,759–12,352)	142.78 (46.31–212.28)	119.76 (27.5–183.01)	−0.56 (−0.6 to −0.53)
Southeast Asia	276,364 (93,707–403,887)	483,022 (111,908–757,919)	119.35 (42.1–176.63)	80.65 (19.08–127.37)	−1.33 (−1.37 to −1.28)
**Sub–Saharan Africa**					
Central Sub-Saharan Africa	21,757 (4,231–34,040)	47,620 (11,146–73,451)	119.67 (28.53–184.66)	111.39 (31.22–169.16)	−0.38 (−0.45 to −0.32)
Eastern Sub-Saharan Africa	75,224 (35,301–103,811)	118,057 (43,975–170,423)	118.93 (61.05–164.22)	84.49 (34.83–122.2)	−1.32 (−1.39 to −1.25)
Southern Sub-Saharan Africa	17,455 (6,311–23,970)	38,165 (15,956–51,825)	71.41 (26.94–100)	77.07 (33.73–105.95)	0.28 (−0.13 to– 0.69)
Western Sub-Saharan Africa	66,064 (15,184–103,082)	115,928 (21,789–180,200)	87.1 (20.96–136.91)	70.03 (13.4–109.94)	−0.84 (−0.96 to −0.72)
**Subgroup**					
Ischemic heart disease	2,628,590 (102,650–4,032,758)	3,906,345 (−63,184–6,213,425)	75.05 (3.06–116.86)	46.76 (−0.77–74.74)	−1.65 (−1.71 to −1.6)
Stroke	881,329 (388,165–1,458,221)	1,077,762 (443,142–1,927,861)	23.52 (9.82–39.66)	12.67 (5.01–22.84)	−2.24 (−2.35 to −2.14)
Hypertensive heart disease	503,985 (397,025–590,272)	824,646 (665,738–999,014)	14.59 (11.61–17.03)	10.08 (8.15–12.21)	−1.1 (−1.23 to −0.98)
Atrial fibrillation and flutter	3,350 (385–10,222)	9,529 (782–30,753)	0.12 (0.01–0.36)	0.12 (0.01–0.39)	0.08 (0.04 to 0.12)
Aortic aneurysm	7,307 (5,309–9,376)	11,127 (8,043–14,582)	0.21 (0.15–0.27)	0.13 (0.1–0.18)	−1.76 (−1.88 to −1.64)
Lower extremity peripheral arterial disease	3,793 (885–7,612)	4,443 (1,129–9,043)	0.12 (0.03–0.25)	0.06 (0.01–0.11)	−3.08 (−3.28 to −2.88)

CVD-DR, cardiovascular disease due to dietary risks; ASMR, age-standardized mortality rate; EAPC, estimated annual percentage change.

**TABLE 2:** DALYs and ASDRs of CVD-DR and its temporal trends from 1990 to 2021.

Location	DALYs (95% UI)		ASDR (95% UI)		1990–2021
	1990	2021	1990	2021	EAPCs (95% CI)
Global	98,331,777 (27,092,345–136,036,264)	134,179,728 (32,581,870–192,859,591)	2,487.47 (675.52–3,480.84)	1,563.86 (378.95–2,246.75)	−1.65 (−1.71 to −1.59)
**SDI**					
High SDI	18,457,746 (3,705,341–26,986,173)	13,968,844 (4,388,989–20,347,991)	1,700.34 (345.5–2,473.78)	704.13 (226.17–999.13)	−2.92 (−3.02 to −2.81)
High-middle SDI	27,128,765 (7,424,835–37,564,381)	29,443,067 (7,660,632–43,146,296)	2,809.86 (753.73–3,930.95)	1,516.27 (385.06–2,215.68)	−2.39 (−2.65 to −2.13)
Middle SDI	27,727,929 (11,109,467–37,670,588)	45,198,388 (12,193,509–64,945,784)	2,654.84 (1,123.96–3,616.56)	1,695.16 (471.77–2,454.79)	−1.49 (−1.53 to −1.45)
Low-middle SDI	18,376,943 (3,530,534–26,085,918)	33,815,791 (5,036,809–49,426,281)	2,816.47 (583.35–4,024.54)	2,260.27 (363.47–3,324.9)	−0.68 (−0.72 to −0.64)
Low SDI	6,495,986 (1,682,512–9,179,713)	11,622,954 (2,856,232–16,501,397)	2,744.59 (753.19–3,910.52)	2,180.08 (606.6–3,116.26)	−0.83 (−0.91 to −0.76)
**Central Europe, Eastern Europe, and Central Asia**					
Central Asia	2,099,157 (355,594–2,881,622)	2,509,208 (237,100–3,633,333)	4,537.62 (760.82–6,289.97)	3,199.1 (308.38–4,689.7)	−1.74 (−2.12 to −1.37)
Central Europe	5,906,938 (1,905,433–8,033,553)	4,098,173 (1,559,235–5,852,199)	4,089.97 (1,325.36–5,565.83)	1,854.04 (676.31–2,625.75)	−2.89 (−3 to −2.78)
Eastern Europe	10,863,487 (1,401,781–15,635,879)	9,320,984 (313,842–14,516,714)	4,039.58 (500.35–5,814.53)	2,718.47 (108.49–4,168.11)	−2.05 (−2.62 to −1.48)
**High income region**					
Australasia	358,243 (−58,960–578,682)	210,195 (−14,315–337,076)	1,552.72 (−257.84–2,496.76)	398.7 (−28.15–622.16)	−4.6 (−4.76 to −4.43)
High-income Asia Pacific	1,931,568 (824,820–2,855,186)	1,588,481 (622,428–2,462,603)	992.91 (422.23–1,471.13)	352.13 (140.11–527.71)	−3.4 (−3.51 to −3.29)
High-income North America	5,785,329 (1,483,053–8,495,944)	5,282,372 (2,362,589–7,272,171)	1,695.66 (442.94–2,461.16)	869.57 (404.99–1,171.73)	−2.19 (−2.29 to −2.09)
Southern Latin America	925,198 (286,801–1,240,820)	676,037 (229,782–928,574)	2,049.2 (633.57–2,752.43)	777.91 (261.46–1,064.71)	−2.91 (−3.07 to −2.75)
Western Europe	8,373,030 (695,941–12,712,006)	4,645,954 (1,045,882–6,976,301)	1,484.82 (123.55–2,216.76)	487.56 (103.94–711.25)	−3.68 (−3.78 to −3.57)
**Latin America and Caribbean**					
Andean Latin America	292,019 (87,590–412,490)	431,016 (107,739–654,631)	1,382.32 (415.26–1,974.71)	722.84 (183.02–1,101.45)	−2.25 (−2.5 to −2)
Caribbean	571,104 (114,493–850,166)	749,613 (242,673–1,119,421)	2,203.25 (440.35–3,297.62)	1,397.93 (454.29–2,085.38)	−1.49 (−1.7 to −1.29)
Central Latin America	1,294,677 (318,811–1,841,614)	2,676,179 (482,849–3,997,771)	1,539.96 (389.66–2,228.04)	1,065.52 (194.34–1,595.4)	−1.44 (−1.69 to −1.19)
Tropical Latin America	1,869,795 (1,040,410–2,510,576)	2,104,102 (1,126,614–2,879,473)	1,971.9 (1,055.93–2,702.63)	812.66 (434.75–1,115.37)	−2.89 (−3.01 to −2.77)
**North Africa and Middle East**					
North Africa and Middle East	6,487,604 (511,912–9,443,052)	10,437,733 (437,331–16,255,573)	3,696.62 (345.76–5,453.95)	2,195.02 (123.74–3,491.65)	−1.83 (−1.88 to −1.77)
**South Asia**					
South Asia	17,127,482 (1,850,737–24,611,471)	36,590,984 (4,313,495–53,694,677)	2,699.21 (374.49–3,942.77)	2,371.53 (303.34–3,499.9)	−0.36 (−0.41 to −0.32)
**Southeast Asia, East Asia, and Oceania**					
East Asia	21,615,961 (12,173,084–29,555,054)	31,293,111 (13,722,441–46,544,358)	2,599.69 (1,454.63–3,578.4)	1,493.76 (635.02–2,227.26)	−1.71 (−1.8 to −1.62)
Oceania	111,867 (27,878–163,216)	248,286 (41,523–374,720)	3,455.38 (1,097.59–5,126.43)	2,936.67 (635.87–4,436.32)	−0.51 (−0.55 to −0.47)
Southeast Asia	7,827,354 (2,396,944–11,222,998)	12,728,022 (2,481,794–19,667,619)	2,881.85 (951.7–4,178.82)	1,883.77 (388.18–2,934.24)	−1.45 (−1.48 to −1.41)
**Sub–Saharan Africa**					
Central Sub-Saharan Africa	610,364 (107,520–944,520)	1,297,994 (260,850–1,970,713)	2,677.62 (558.81–4,150.02)	2,355.75 (577.5–3,600.92)	−0.59 (−0.66 to −0.51)
Eastern Sub-Saharan Africa	2,075,695 (899,604–2,840,525)	3,196,732 (1,090,769–4,539,272)	2,706.28 (1,263.86–3,721.45)	1,830.02 (675.86–2,644.59)	−1.52 (−1.6 to −1.43)
Southern Sub-Saharan Africa	480,197 (164,194–642,365)	988,212 (395,948–1,335,783)	1,669.55 (588.29–2,274.88)	1,679.48 (685.10–2,282.81)	0.06 (−0.35 to 0.48)
Western Sub-Saharan Africa	1,724,707 (393,711–2,636,883)	3,106,340 (579,225–4,707,242)	1,935.18 (448.56–3,004.54)	1,519.47 (290.77–2,356.01)	−0.91 (−1.04 to −0.79)
**Subgroup**					
Ischemic heart disease	6,337,7588 (1,023,216–93,805,450)	89,929,809 (−2,083,545–137,501,226)	1,608.14 (37.45–2,404.74)	1,049.02 (−24.85–1,604.86)	−1.54 (−1.61 to −1.47)
Stroke	23,413,718 (11,356,988–36,851,530)	27,379,104 (13,000,767–45,753,348)	577.56 (270.67–924.56)	315.53 (147.42–529.71)	−2.19 (−2.28 to −2.09)
Hypertensive heart disease	11,180,596 (8,620,607–13,058,403)	16,249,030 (13,430,389–19,269,003)	291.95 (228.01–340.84)	191.98 (158.54–227.78)	−1.32 (−1.45 to −1.19)
Atrial fibrillation and flutter	114,175 (15,295–328,546)	282,457 (32,790–846,886)	3.23 (0.4–9.53)	3.36 (0.38–10.13)	0.1 (0.09 to 0.12)
Aortic aneurysm	156,111 (114,851–201,069)	232,454 (169,343–302,761)	4.05 (2.96–5.21)	2.72 (1.98–3.54)	−1.61 (−1.72 to −1.49)
Lower extremity peripheral arterial disease	89,589 (23,165–174,693)	106,874 (30,328–212,727)	2.55 (0.65–4.96)	1.27 (0.36–2.52)	−2.76 (−2.93 to −2.59)

CVD-DR, cardiovascular disease due to dietary risks; DALYs, disability-adjusted life years; ASDR, age-standardized DALY rate; EAPC, estimated annual percentage change.

**FIGURE 1:**
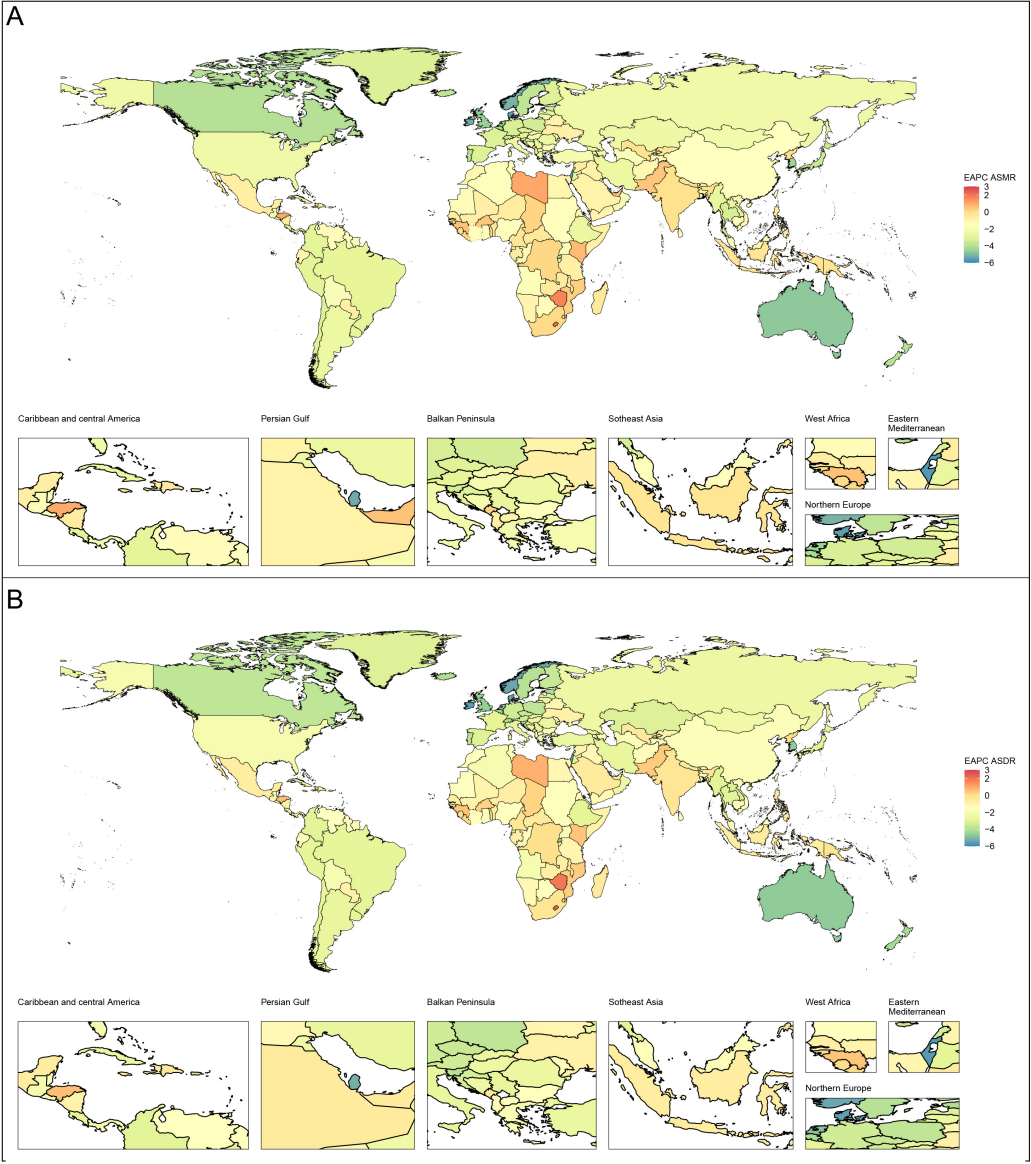
Global EAPCs of CVD-DR in 204 countries or territories from 1990 to 2021. **(A)** EAPC of ASMR; **(B)** EAPC of ASDR. CVD-DR, cardiovascular disease due to dietary risks; EAPCs, estimated annual percentage changes; ASMR, age-standardized mortality rate; ASDR, age-standardized disability-adjusted life years rate.

**FIGURE 2:**
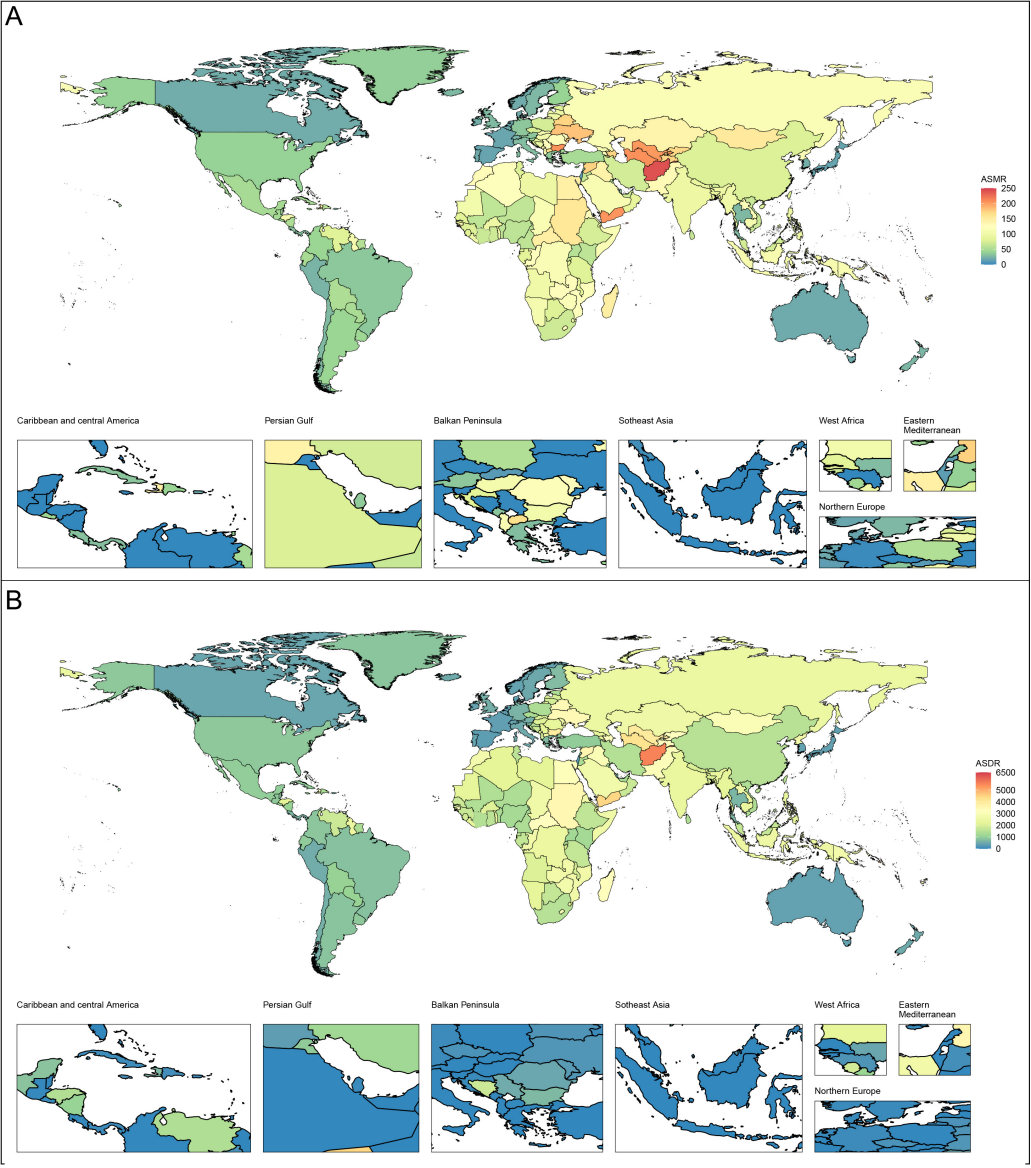
Age-standardized rates of CVD-DR in 204 countries or territories in 2021. **(A)** ASMR; **(B)** ASDR. CVD-DR, cardiovascular disease due to dietary risks; ASMR, age-standardized mortality rate; ASDR, age-standardized disability-adjusted life years rate.

In 2021, the top five countries or regions with the highest ASMR per 100,000 population were Afghanistan (240.75, 95% UI 53.43–362.41), Nauru (239.81, 95% UI 29.75–389.13), Turkmenistan (206.33, 95% UI 18.13–326.28), the Republic of Bulgaria (205.53, 95% UI 112.44–282.34), and Yemen (204.16, 95% UI 39.24–320.67). The top five countries or regions with the highest ASDR were Nauru (6,306.6, 95% UI 340.04–10,244.29), Afghanistan (5,572.81, 95% UI 995.75–8,294.18), the Solomon Islands (4,868.11, 95% UI 778.61–7,788.82), the Republic of the Marshall Islands (4,770.32, 95% UI 824.67–7,537.29), and the Federated States of Micronesia (4,698.56, 95% UI 830.95–7,596.34). Many of these countries or regions are low- or middle-income and face additional challenges, including political instability, economic hardship, and limited public health capacity. The burden and trends of CVD-DR across 204 countries and territories are shown in Figures 1, 2.

#### 3.1.2 CVD-DR subtypes

From 1990 to 2021, only the ASMR and ASDR of atrial fibrillation and flutter due to dietary risks (AFF-DR) increased, with EAPCs of 0.08 (95% CI 0.04–0.12) and 0.1 (95% CI 0.09–0.12), respectively. This trend indicates a growing health burden associated with AFF- DR, underscoring the limitations of current public health manage- ment efforts in addressing this issue. In contrast, the ASDR and ASMR of ischemic heart disease due to dietary risk (IHD-DR), stroke due to dietary risk (stroke-DR), hypertensive heart disease due to dietary risk (HHD-DR), aortic aneurysm due to dietary risk (AA-DR), and lower-extremity peripheral artery disease due to dietary risk (LEPAD-DR) decreased. These findings suggest that global dietary interventions, public health measures, and improvements in healthcare have helped reduce the burden of these conditions. In 2021, the ASDR and ASMR of IHD-DR were the highest among the six subtypes, at 1,049.02 (95% UI −24.85 to 1,604.86) and 46.76 (95% UI −0.77 to 74.74), highlighting the significant ongoing contribution of poor diet to ischemic heart disease and emphasizing the need for continued preventive efforts.

The highest ASMR in 2021 were observed in the Republic of Uzbekistan for IHD-DR (181.03, 95% UI −0.55 to 282.43 per 100,000 population), North Macedonia for stroke-DR (50.93, 95% UI 14.65–90.69 per 100,000 population), the Republic of Bulgaria for HHD-DR (64.15, 95% UI 47.33–81.29 per 100,000 population), Montenegro for AFF-DR (1.14, 95% UI 0.19–2.71 per 100,000 population), Nauru for AA-DR (0.59, 95% UI 0.33–0.97 per 100,000 population), and the Republic of Belarus for LEPAD-DR (0.33, 95% UI 0.06–0.70 per 100,000 population). The highest age-standardized disability rates (ASDR) were recorded for Nauru for IHD-DR (4,748.75, 95% UI −996.13 to 8,341.30 per 100,000 population), the Solomon Islands for stroke-DR (1,255.08, 95% UI 370.03–2,120.10 per 100,000 population), Lesotho for HHD-DR (1,169.27, 95% UI 624.60–1,656.31 per 100,000 population), Montenegro for AFF-DR (17.76, 95% UI 3.18–40.61 per 100,000 population), Nauru for AA-DR (11.85, 95% UI 7.64–17.30 per 100,000), and the Republic of Belarus for LEPAD-DR (6.64, 95% UI 1.31–14.13 per 100,000 population). These data indicate that certain countries face disproportionately high burdens from specific CVD subtypes, which may be influenced by local diet patterns, healthcare access, and genetic predispositions. Tables 1, 2 summarize the burden and temporal trends of CVD-DR subtypes.

### 3.2 SDI differences

#### 3.2.1 CVD-DR

**FIGURE 3:**
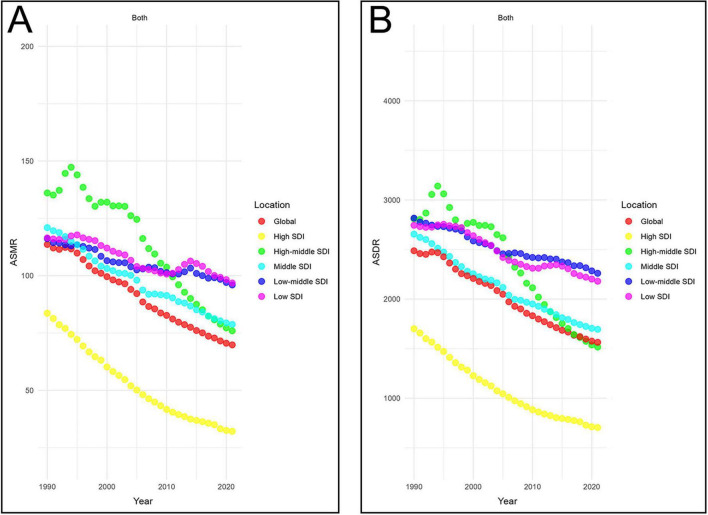
Temporal trends in age-standardized rates of CVD-DR across SDI quintiles. **(A)** ASMR; **(B)** ASDR. CVD-DR, cardiovascular disease due to dietary risks; SDI, socio-demographic index; ASMR, age-standardized mortality rate; ASDR, age-standardized disability-adjusted life years rate.

From 1990 to 2021, both the ASDR and ASMR decreased across all SDI regions. The steepest declines were observed in high-SDI regions, with EAPCs of −3.19 (95% CI −3.28 to −3.1) for ASMR and −2.92 (95% CI −3.02 to −2.81) for ASDR. In contrast, the slowest declines were observed in low-middle SDI regions, with EAPCs of −0.57 (95% CI −0.62 to −0.51) for ASMR and −0.68 (95% CI −0.72 to −0.64) for ASDR. In 2021, low-SDI regions had the highest ASMR (96.81, 95% UI 30.01–139.76 per 100,000 population), while low-middle SDI regions had the highest ASDR (2,260.27, 95% UI 363.47–3,324.9 per 100,000 population) (Figure 3). These findings indicate that low and low-middle SDI regions not only face the heaviest burdens of CVD-DR but also show the slowest progress in reducing it.

**FIGURE 4:**
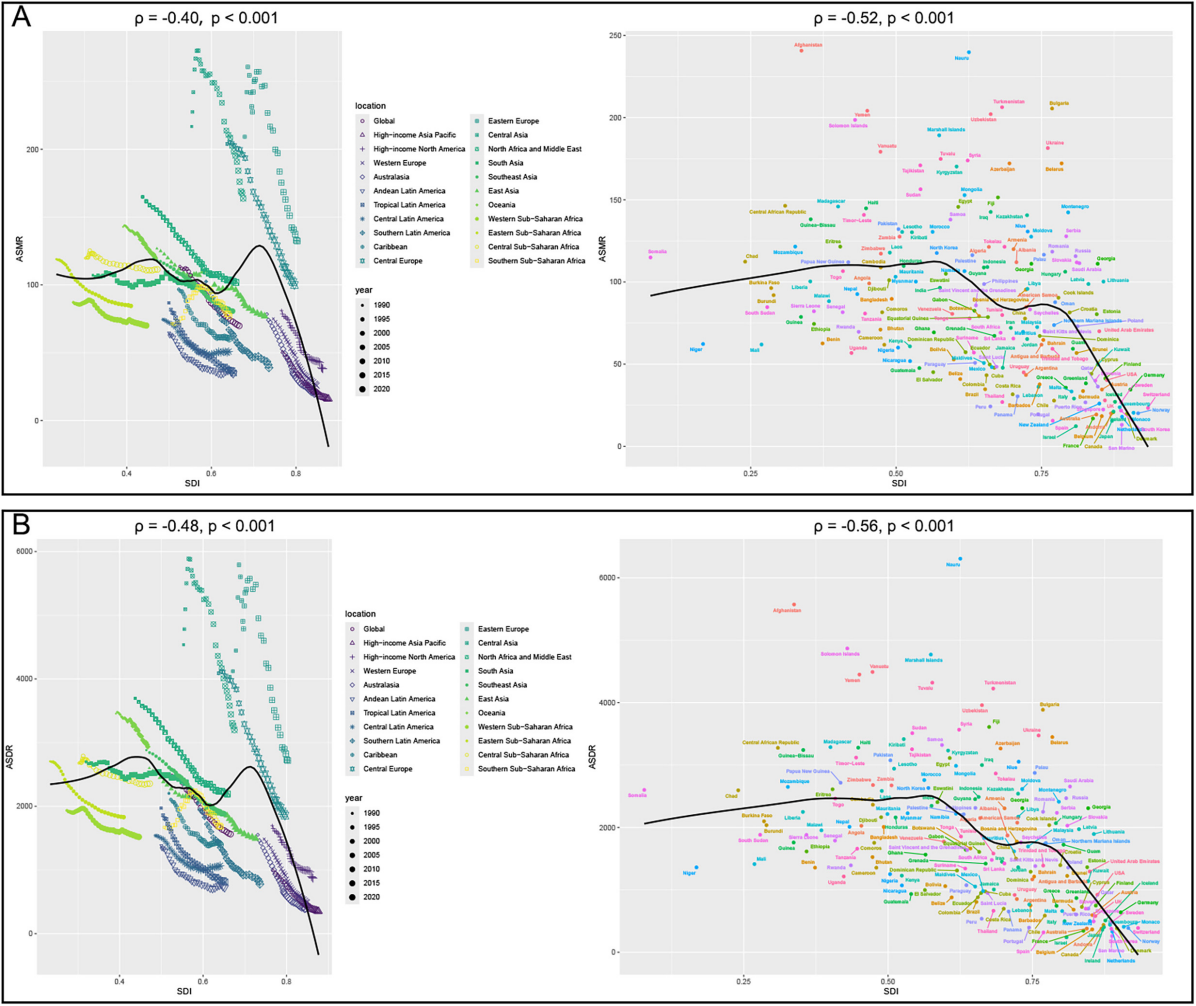
Correlation analyses between age-standardized rates of CVD-DR and SDI from 1990 to 2021. **(A)** ASMR; **(B)** ASDR. CVD-DR, cardiovascular disease due to dietary risks; SDI, socio-demographic index; ASMR, age-standardized mortality rate; ASDR, age-standardized disability-adjusted life years rate.

**FIGURE 5:**
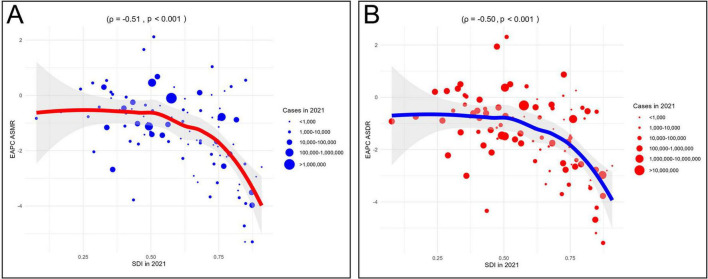
Correlation analyses between EAPCs of CVD-DR and SDI in 2021. **(A)** EAPC of ASMR; **(B)** EAPC of ASDR. CVD-DR, cardiovascular disease due to dietary risks; SDI, socio-demographic index; ASMR, age-standardized mortality rate; ASDR, age-standardized disability-adjusted life years rate; EAPCs, estimated annual percentage changes.

Correlation analysis further supported these patterns. ASMR was negatively correlated with SDI at both the 22 regions (22 regions, ρ = −0.40, *P* < 0.001) and 204 countries/territories (ρ = −0.52, *p* < 0.001) (Figure 4A). Similarly, ASDR was inversely correlated with SDI in 22 regions (ρ = −0.48, *p* < 0.001) and 204 countries/territories (ρ = −0.56, *P* < 0.001) (Figure 4B). Additionally, the EAPCs of ASDR (ρ = −0.51, *p* < 0.001) and ASMR (ρ = −0.50, *p* < 0.001) were negatively correlated with SDI in 2021 (Figure 5). Taken together, these analyses demonstrate that regions with lower SDI bear a greater burden of CVD-DR and achieve slower reductions over time.

#### 3.2.2 CVD-DR subtypes

From 1990 to 2021, the ASMR and ASDR of IHD-DR and stroke-DR decreased in all five SDI regions, with the slowest decline in the low-SDI regions (Supplementary Figures 1A, B). In contrast, HHD-DR showed rising ASMR and ASDR only in high-SDI regions, while all other regions experienced declines (Supplementary Figure 1C). For AFF-DR, both rates increased in nearly all regions except the middle-SDI group, with the steepest growth occurring in low-middle and low-SDI regions (Supplementary Figure 1D). Similarly, AA-DR and LEPAD-DR rose exclusively in low-middle and low-SDI regions (Supplementary Figures 1E, F). Collectively, these findings suggest that low and low-middle SDI regions face increasing burdens from IHD-DR, stroke-DR, AFF- DR, AA-DR, and LEPAD-DR, whereas high-SDI regions are primarily affected by rising HHD-DR.

Correlation analysis further supported these patterns. The ASDR and ASMR of IHD-DR, stroke-DR, and HHD-DR were negatively correlated with SDI (ρ < 0), whereas those of AFF-DR, AA-DR, and LEPAD-DR showed positive correlations with SDI (ρ > 0). These correlations were statistically significant in at least one of the 22 regions and 204 countries/territories (*p* < 0.05; Supplementary Figure 2). Additionally, the EAPCs of ASMR and ASDR for IHD-DR, stroke-DR, AFF-DR, and AA-DR were negatively correlated with SDI (ρ < 0; Supplementary Figure 3). These results underscore the heterogeneity in the associations between different CVD subtypes and SDI and highlight that the burden of major subtypes, particularly IHD-DR, stroke-DR, and HHD-DR, remains disproportionately higher in lower-SDI regions.

### 3.3 Sex and age differences

#### 3.3.1 CVD-DR

**FIGURE 6:**
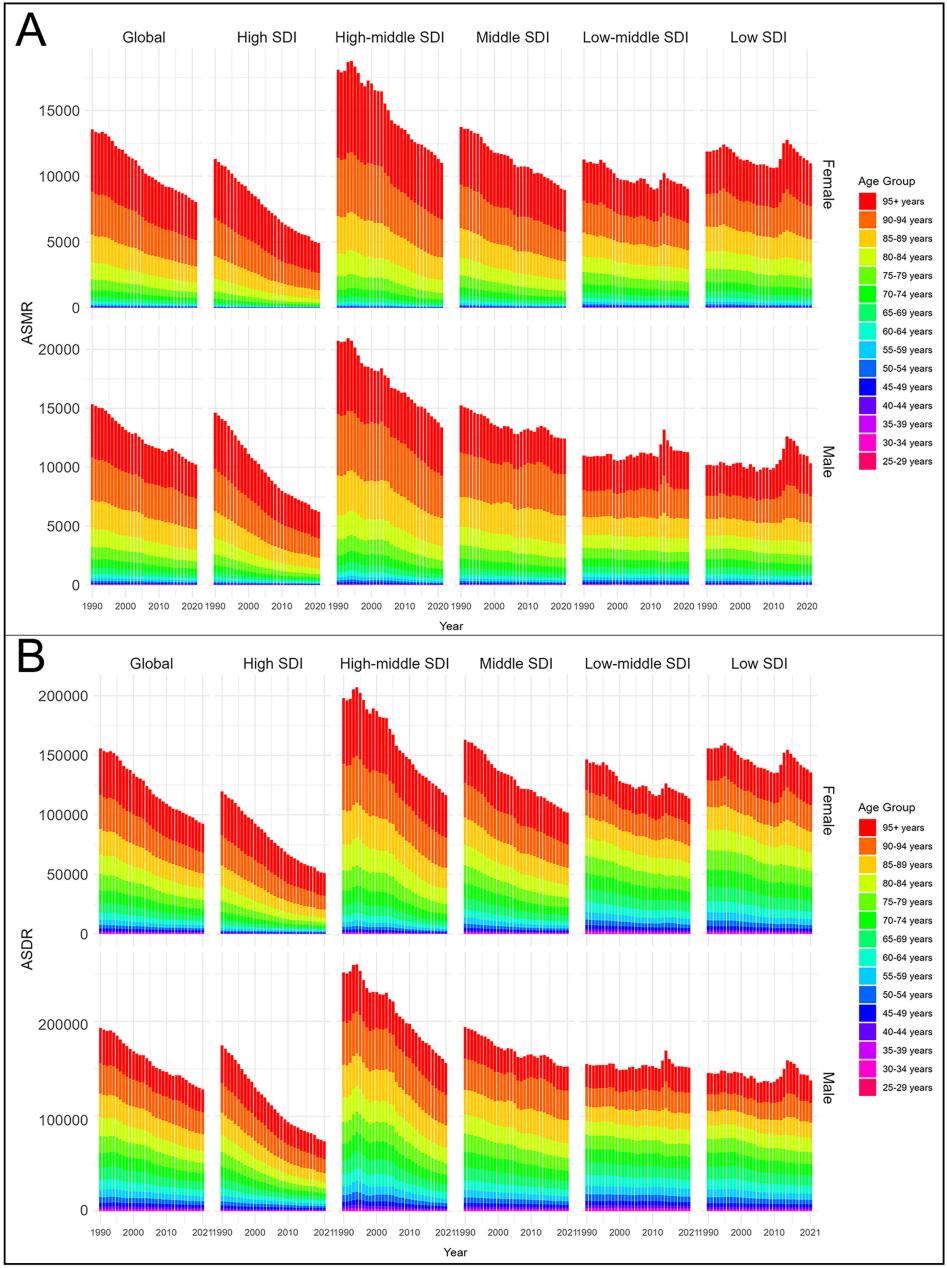
Age-standardized rates of CVD-DR by sex and age group from 1990 to 2021. **(A)** ASMR; **(B)** ASDR. CVD-DR, cardiovascular disease due to dietary risks; ASMR, age-standardized mortality rate; ASDR, age-standardized disability-adjusted life years rate.

From 1990 to 2021, both ASMR and ASDR declined in females and males. In 2021, males had higher values than females, with an ASMR of 87.59 (95% UI 21.87–128.64) and an ASDR of 2,020.24 (95% UI 465.45–2,889.14) per 100,000 population, compared with an ASMR of 54.61 (95% UI 13.42–83.33) and an ASDR of 1,143.99 (95% UI 284.15–1,690.92) in females. However, these simple numerical comparisons were based solely on GBD estimates, and statistical tests could not be conducted to confirm the significance of these differences. SDI-stratified analyses further showed that ASMR and ASDR declined more slowly in low and low-middle SDI regions for both sexes. In 2021, both indicators remained higher in the low SDI and low-middle SDI groups for both sexes (Figure 6). These findings indicate that males consistently carry a greater burden of CVD-DR than females, while low and low-middle SDI regions face both higher burdens and slower declines across sexes.

From 1990 to 2021, both ASMR and ASDR declined across all age groups, although rates in the 95+ year age group remained consistently higher than in younger age groups. Among those aged ≥95+ years, the ASMR and ASDR were 2,864.01 (95% UI 797.87–4,487.51) and 23,884.53 (95% UI 6,702.42–37,348.44) per 100,000 population, respectively, for males, and 2,905.88 (95% UI 829.07–4,569.62) and 24,052.24 (95% UI 7,002.67–37,966.46) per 100,000 population for females. SDI-stratified analyses further showed that adults aged 25–59 years in low and low-middle SDI regions had substantially higher ASMR and ASDR than their counterparts in higher-SDI regions (Figure 6). These findings highlight advanced age as the strongest determinant of CVD-DR burden, while also underscoring the disproportionate risks faced by younger adults in low-SDI regions.

#### 3.3.2 CVD-DR subtypes

From 1990 to 2021, the ASDR and ASMR of AFF-DR increased in both males and females, whereas the rates for other subtypes decreased. In 2021, males showed higher ASMR and ASDR for IHD-DR, stroke-DR, AFF-DR, and AA-DR, whereas females showed higher rates for HHD-DR and LEPAD-DR. These patterns demonstrate the heterogeneity of sex differences in CVD subtypes, with males experiencing greater burdens of IHD- DR, stroke-DR, AFF-DR, and AA-DR, and females bearing a higher burden of HHD-DR and LEPAD-DR.

Age-stratified trends from 1990 to 2021 revealed that the ASMR and ASDR of AFF-DR were stable or increased across all age groups, whereas the rates for other subtypes generally decreased. In 2021, the ASDR and ASMR of each CVD-DR subtype were concentrated among individuals aged ≥65 years, increasing progressively with age, similar to the overall CVD-DR trend (Supplementary Figure 4). These findings highlight that advanced age remains the primary risk factor for CVD-DR subtypes.

### 3.4 Dietary risk factor analysis

The GBD 2021 data reported 13 dietary risks associated with CVD, including low intake of fruits, vegetables, whole grains, nuts and seeds, seafood omega-3 fatty acids, fiber, legumes, and polyunsaturated fatty acids, vegetables, as well as high intake of sodium, processed meat, trans fatty acids, red meat, and sugar-sweetened beverages. In 2021, the highest percentages of deaths and DALYs were attributable to diets low in whole grains, diets low in fruits, and diets high in sodium, thereby identifying these as the primary dietary risks contributing to the burden of CVD-DR.

#### 3.4.1 Diet high in sodium

**FIGURE 7:**
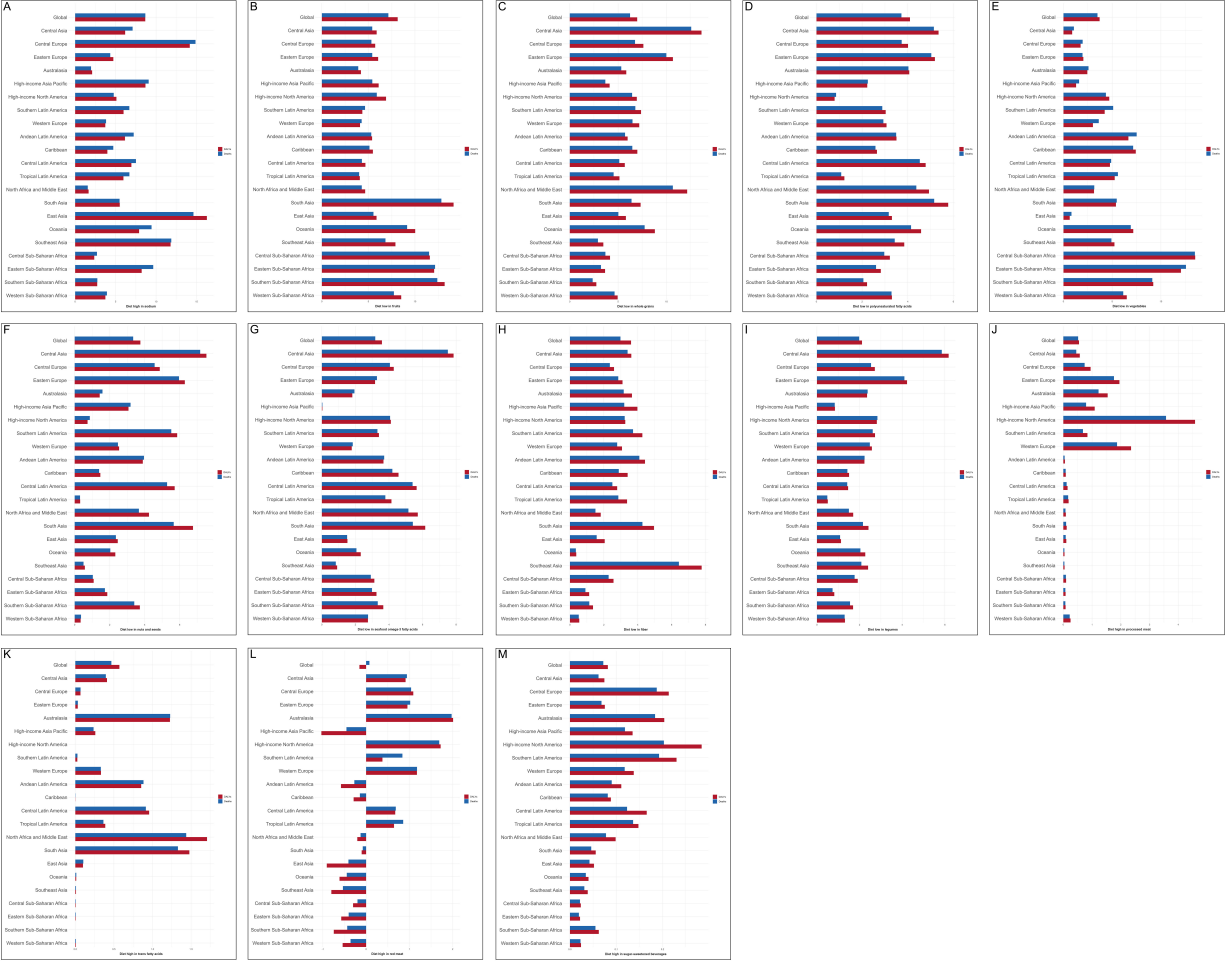
Thirteen dietary risks contributing to deaths and DALYs attributable to CVD in 2021. These included diets **(A)** high in sodium, **(B)** low in fruits, **(C)** low in whole grains, **(D)** low in polyunsaturated fatty acids, **(E)** low in vegetables, **(F)** low in nuts and seeds, **(G)** low in seafood-derived omega-3 fatty acids, **(H)** low in fiber, **(I)** high in processed meat, **(J)** low in legumes, **(K)** high in trans fatty acids, **(L)** high in red meat, and **(M)** high in sugar-sweetened beverages. CVD-DR, cardiovascular disease due to dietary risks; DALYs, disability-adjusted life years.

Globally, CVD attributable to diet high in sodium accounted for 8.67% of total deaths and 8.66% of total DALYs. At the regional level, Central Europe exhibited the highest proportion of deaths (14.87%), while East Asia showed the highest proportion of DALYs (16.28%), as illustrated in Figure 7A. Moreover, diet high in sodium was the leading contributor to the burden of stroke-DR (deaths 9.94%; DALYs 10.63%) and AFF-DR (deaths 2.77%; DALYs 3.32%), as shown in Supplementary Figures 5, 6. These findings indicate that Central Europe and East Asia face particularly heavy burdens from excessive sodium intake, and that high sodium consumption is a major dietary risk factor for stroke and AFF.

#### 3.4.2 Diet low in fruits

Globally, CVD attributable to diet low in fruits accounted for 7.14% of total deaths and 8.14% of total DALYs. At the regional level, South Asia had the highest proportions, with deaths at 12.83% and DALYs at 14.15%, as illustrated in Figure 7B. Moreover, diet low in fruits was the most significant contributor to the burden of HHD-DR (deaths 36.0%; DALYs 37.8%) and AA-DR (deaths 3.63%; DALYs 3.77%), as shown in Supplementary Figures 7, 8. These findings suggest that South Asia bears the greatest burden from insufficient fruit consumption, and that low fruit intake is a major dietary risk factor for HHD and AA.

#### 3.4.3 Diet low in whole grains

Globally, CVD attributable to diet low in whole grains accounted for 6.24% of total deaths and 6.97% of total DALYs. At the regional level, Central Asia had the highest proportions, with deaths at 12.57% and DALYs at 13.63%, as illustrated in Figure 7C. Moreover, diet low in whole grains was the leading contributor to the burden of IHD-DR (deaths 12.4%; DALYs 14.3%), as shown in Supplementary Figure 9. These findings indicate that Central Asia experiences the highest burden from insufficient whole grain intake, and that low whole grain consumption is a major dietary risk factor for IHD.

#### 3.4.4 Diet low in polyunsaturated fatty acids

Globally, CVD attributable to diet low in polyunsaturated fatty acids accounted for 3.72% of total deaths and 4.10% of total DALYs. At the regional level, South Asia had the highest proportions, with deaths at 5.16% and DALYs at 5.77%, as illustrated in Figure 7D. These findings suggest that South Asia is disproportionately affect- ed by insufficient intake of polyunsaturated fatty acids.

#### 3.4.5 Diet low in vegetables

Globally, CVD attributable to diet low in vegetables accounted for 3.49% of total deaths and 3.71% of total DALYs. At the regional level, Central Sub-Saharan Africa had the highest burden, with deaths at 13.52% and DALYs at 13.48%, as shown in Figure 7E. These findings indicate that Central Sub-Saharan Africa experienc- es the greatest burden from inadequate vegetable consumption.

#### 3.4.6 Diet low in nuts and seeds

Globally, CVD attributable to diet low in nuts and seeds accounted for 3.34% of total deaths and 3.75% of total DALYs. At the regional level, Central Asia had the highest proportions, with deaths at 7.17% and DALYs at 7.53%, as illustrated in Figure 7F. These findings indicate that Central Asia faces a substantial CVD burden associated with insufficient consumption of nuts and seeds.

#### 3.4.7 Diet low in seafood omega-3 fatty acids

Globally, CVD attributable to diet low in seafood omega-3 fatty acids accounted for 3.18% of total deaths and 3.58% of total DALYs. Regionally, Central Asia had the highest burden, with deaths at 7.51% and DALYs at 7.85%, as shown in Figure 7G. These findings suggest that Central Asia is the most affected by inadequate intake of seafood-derived omega-3 fatty acids.

#### 3.4.8 Diet low in fiber

Globally, CVD attributable to diet low in fiber accounted for 2.98% of total deaths and 3.61% of total DALYs. At the regional level, Southeast Asia reported the highest proportions, with deaths at 6.43% and DALYs at 7.77%, as illustrated in Figure 7H. These findings indicate that Southeast Asia bears the greatest CVD burden related to insufficient fiber consumption.

#### 3.4.9 Diet high in processed meat

Globally, CVD attributable to diet high in processed meat accounted for 2.10% of total deaths and 2.10% of total DALYs. Regionally, high-income North America exhibited the highest burden, with deaths at 3.90% and DALYs at 4.80%, as shown in Figure 7I. Moreover, diet high in processed meat was the leading contributor to the burden of LEPAD-DR (deaths 2.10%; DALYs 2.10%), as presented in Supplementary Figure 10. These findings suggest that high-income North America is most affected by excessive processed meat intake, and that high processed meat consumption is a major dietary risk factor for LEPAD.

#### 3.4.10 Diet low in legumes

Globally, CVD attributable to diet low in legumes accounted for 1.98% of total deaths and 2.11% of total DALYs. At the regional level, Central Asia exhibited the highest burden, with deaths at 5.85% and DALYs at 6.18%, as illustrated in Figure 7J. These findings indicate that Central Asia experiences the greatest CVD burden associated with insufficient legume consumption.

#### 3.4.11 Diet high in trans fatty acids

Globally, CVD attributable to diet high in trans fatty acids accounted for 0.48% of total deaths and 0.57% of total DALYs. Regionally, the highest proportions were observed in North Africa (death 1.44%) and the Middle East (DALYs 1.71%), as shown in Figure 7K. These findings suggest that North Africa and the Middle East bear the greatest CVD burden related to excessive trans fatty acid intake.

#### 3.4.12 Diet high in sugar-sweetened beverages

Globally, CVD attributable to diet high in sugar-sweetened beverages accounted for 0.07% of total deaths and 0.08% of total DALYs. At the regional level, high-income North America had the highest proportions, with deaths at 0.20% and DALYs at 0.28%, as illustrated in Figure 7L. These findings indicate that high-income North America faces the highest CVD burden associated with sugar-sweetened beverage consumption.

#### 3.4.13 Diet high in red meat

Globally, CVD attributable to diet high in red meat accounted for 0.08% of total deaths and 0.15% of total DALYs. Regionally, Australasia exhibited the highest burden, with deaths at 1.98% and DALYs at 2.01%, as shown in Figure 7M. These findings suggest that Australasia bears the greatest CVD burden linked to high red meat intake.

## 4 Discussion

### 4.1 Research significance and main findings

This study offers the first comprehensive assessment of the burden of CVD-DR, including global trends and their influencing factors. The key findings are as follows: First, between 1990 and 2021, the absolute numbers of deaths and DALYs attributable to CVD-DR increased worldwide, whereas the ASMR and ASDR showed declines. IHD-DR, stroke-DR, and HHD-DR were the primary contributors to the worldwide CVD-DR burden. Second, both ASMR and ASDR were negatively correlated with SDI. Third, the EAPCs of ASMR and ASDR also demonstrated negative correlations with SDI. Fourth, the ASMR and ASDR increased with age and remained higher in males than in females. Fifth, high sodium intake, low fruit intake, and low whole grains consumption were the leading dietary contributors to the global CVD-DR burden. In the following section, we discuss these findings in the context of existing literature.

### 4.2 Global and regional burden analysis

Over the past 31 years, although global DALYs and deaths from CVD-DR have increased by 44.8 and 36.5%, respectively, the ASDR and ASMR have declined to 61.4 and 62.9% of their 1990 values, respectively. This apparent discrepancy is primarily attributable to rapid global population growth, which has increased the absolute number of cases despite reductions in age-standardized rates. A previous study based on the GBD 2019 data reported a similar pattern, noting an overall increase in the burden of CVD-DR despite a decline in ASR, which the authors also attributed to population growth (14). The trends in death, DALYs, ASMR, and ASDR for IHD-DR, stroke-DR, HHD-DR, AA-DR, and LEPAD-DR were the same as those for CVD-DR, but increased for AFF-DR. In 2021, IHD-DR, stroke-DR, and HHD-DR were the main sources of CVD-DR burden, accounting for 67.0, 18.2, and 14.4% of the ASMR and 67.7, 20.2, and 12.3% of the ASDR, respectively. However, AFF-DR, AA-DR, and LEPAD-DR together accounted for only 0.4 and 0.5% of ASMR and ASDR, respectively. These findings underscore the persistent significant health burden of CVD-DR, particularly for patients with IHD-DR, stroke-DR, and HHD-DR.

In 2021, Central Asia showed the highest ASMR and ASDR, whereas high-income regions such as Western Europe, Southern Latin America, high-income North America, high-income Asia Pacific, and Australasia had significantly lower CVD-DR burdens, suggesting that socioeconomic level is an important influencing factor of CVD-DR burden. Additionally, at the country level, Afghanistan, Nauru, Turkmenistan, Bulgaria, Yemen, the Solomon Islands, the Marshall Islands, and the Federated States of Micronesia had the highest CVD-DR burdens, highlighting the need for more aggressive prevention and treatment policies. Notably, Southern Sub-Saharan Africa was the only region where ASMR and ASDR increased from 1990 to 2021, suggesting that it may emerge as a major hotspot for CVD-DR. This upward trend is likely driven by dietary shifts associated with rapid urbanization and globalization. Since the mid-1990s, following the Southern African Development Community’s implementation of trade and economic liberalization, local diets have transitioned from traditional, fiber- and nutrient-rich foods to imported, ultra-processed products, and sugar-sweetened beverages high in salt, sugar, and fat (15). Concurrently, population growth and increased life expectancy have increased the number of individuals with elevated cardiovascular risk, whereas limited preventive strategies and inadequate acute care capacity have further contributed to rising mortality (16, 17). Unlike most regions where ASMR and ASDR declined over the same period, this divergence highlights the urgency and complexity of CVD-DR prevention in Southern Sub-Saharan Africa. Conversely, Australasia experienced the steepest decline in ASDR and ASMR between 1990 and 2021. Although we also observed declines in high-income Western countries in Europe and North America, the reduction was most pronounced in Australasia. This difference may reflect sustained policy-driven improvements in dietary patterns in this region. Since the early 21st century, federal and state governments have implemented coordinated public health strategies aimed at reducing unhealthy diets and sugar-sweetened beverage consumption. For example, in 2012, Western Australia introduced the *Western Australian Health Promotion Strategic Framework 2017–2021*, which emphasized balanced diets and reduced consumption of unhealthy foods and sugary drinks (18). Similarly, in 2015, the Australian government launched the Healthy Food Partnership and in 2018 proposed nutritional reformulation targets to reduce consumption of sugar in beverages by 20% by 2025 (19). These policy initiatives have played a significant role in improving dietary behaviors within the region and may have contributed to the observed reductions in the ASMR and ASDR of CVD-DR.

Overall, the CVD-DR burden in a given region is influenced by a combination of factors, including socioeconomic level, dietary patterns, demographics, and healthcare level. Recognizing these regional differences is essential for designing effective, context-specific prevention and management strategies.

### 4.3 Burden analysis based on SDI

Correlation analysis showed that the ASMR and ASDR of CVD-DR and its major subtypes, IHD-DR, stroke-DR, and HHD-DR, were negatively correlated with SDI, revealing a strong association between socioeconomic status and the burden of CVD-DR. Since 2010, the low and low-middle SDI regions have been the primary areas affected by CVD-DR (Figure 3). The burden on these regions may be shaped by the combined effects of socioeconomic status on dietary patterns, health care levels, and chronic stress. First, socioeconomic level determines the average income level of a population, which in turn influences food consumption. In low-SDI regions, low incomes make it difficult for residents to afford healthy and abundant food, thereby increasing the consumption of cheap and unhealthy processed foods (20). However, unhealthy food consumption significantly increases the risks of diabetes, hypertension, obesity, and other metabolic diseases, all of which are important risk factors for CVD (20, 21). Second, socioeconomic level directly influences healthcare expenditure and the quality of medical services in a given region. In low-SDI regions, a weak socioeconomic foundation leads to limited healthcare spending, which further restricts improvements in healthcare levels and the development of healthcare systems. For example, in Nepal, national health insurance coverage does not exceed 10% of the population, > 50% of health expenditure is out-of-pocket, and funding for CVD-related healthcare is minimal (22). Moreover, inadequate healthcare can lead to delayed diagnosis, poor management, and a lack of preventive care. Third, socioeconomic level affects mental mood through income level (23). Residents of low-SDI regions may experience chronic stress due to low incomes, job instability, and social isolation (23). Chronic stress can activate the hypothalamic-pituitary-adrenal axis and autonomic nervous system, leading to obesity, dyslipidemia, and insulin resistance, thereby increasing CVD risk (24). Moreover, chronic stress can induce or exacerbate CVD via chronic inflammation, oxidative stress, and endothelial dysfunction (25, 26). These findings indicate that socioeconomic status influences disease burden through dietary patterns, healthcare levels, and chronic stress, which helps explain the link between CVD-DR burden and SDI.

Notably, from 2010 to 2021, low and low-middle SDI regions showed a hump-shaped pattern in ASMR and ASDR, with rates first increasing and then declining, whereas the high-SDI regions displayed a steady, linear decrease. In low- and low-middle SDI regions, the short-term increase was likely driven by rapid urbanization and dietary shifts, including greater consumption of ultra-processed foods, high-sodium meals, and sugar-sweetened beverages, which temporarily offset the benefits of incremental healthcare improvements (27–29). As healthcare systems strengthened and comprehensive prevention strategies, such as salt-reduction initiatives, the Framework Convention on Tobacco Control, and dietary optimization, were more widely implemented, the CVD-DR burden began to decline (30–32). In contrast, high-SDI regions had already completed urbanization and established relatively stable dietary patterns. Combined with earlier adoption of effective prevention policies and advanced healthcare services, these factors contributed to the consistent and sustained declines in ASMR and ASDR.

Additionally, the growth rates of ASDR and ASMR for CVD-DR, IHD-DR, and stroke-DR were negatively correlated with SDI. High-SDI regions showed the most rapid decline in ASMR and ASDR, which was attributed to their high socioeconomic levels. In such regions, strong economic foundations positively impacted local healthcare systems and dietary management. First, per capita health expenditure and health investment in high and middle-high SDI regions are significantly higher than in other SDI regions, leading to the development of more comprehensive healthcare systems (33). Thus, patients with CVD in high-SDI regions receive more comprehensive and advanced preventive, diagnostic, and treatment services, thereby minimizing the health burden caused by CVD-DR. Moreover, these individuals benefit from favorable economic and educational environments, which allow them to choose healthy diets (34). With economic development between 1999 and 2016, the percentage of energy intake from low-quality carbohydrates among US adults decreased significantly, whereas the intake percentages of polyunsaturated fats, plant proteins, and energy from high-quality carbohydrates increased significantly (35). A subsequent clinical study in Canada reported that individuals who adhered to the Canadian Food Guide had a 24% lower risk of developing CVD compared with those who did not (RR: 0.76; 95% CI 0.58–0.94) (36). In general, the positive impact of socioeconomic level on healthcare systems and dietary management explains the association between CVD-DR burden trends and SDI.

Interestingly, among all the subtypes, only the growth rates of ASMR and ASDR of HHD-DR were positively correlated with SDI, with the highest levels observed in high-SDI regions. The rapid increase in the HHD-DR burden in these regions may be related to increasing aging populations. Aging is among the most important risk factors for hypertension and a direct cause of HHD (37). However, although the burdens of HHD-DR in low-SDI regions decrease with socioeconomic development, these regions show the highest ASDR and ASMR. Thus, low-SDI regions remain the primary hotspots for HHD-DR, and their healthcare systems and dietary patterns require further improvement. In summary, the burden of CVD-DR and its subtypes is intricately linked to the SDI through the interplay of socioeconomic levels, healthcare levels, dietary patterns, and population aging. Thus, effective reduction of CVD-DR burden requires prevention and management strategies that are adapted to regional differences in SDI.

### 4.4 Burden analysis based on sex and age

Sex-stratified analysis revealed that males had higher ASMR and ASDR values for CVD-DR than females, which may be explained by differences in sex hormone levels, lifestyle behaviors, and healthcare utilization. Biologically, males have significantly lower estrogen levels than females, and estrogen exerts protective effects on the cardiovascular system by downregulating plasminogen activator inhibitor-1, inflammatory markers, and matrix metalloproteinases (38). Behaviorally, males are more likely to engage in unhealthy lifestyle habits such as smoking, alcohol consumption, and unhealthy diet, which are important risk factors for CVD. The World Health Organization (WHO) reports significantly higher global rates of alcohol consumption and smoking among males compared with females (52% and 36.7%, respectively, vs. 35% and 7.8%) (39, 40). Dietary assessments similarly indicate that females generally follow healthier dietary patterns, while males consume higher amounts of ultra-processed foods (41, 42). Additionally, males are more likely to work in physically demanding or high-risk occupations such as construction, mining, and manufacturing, which increases exposure to occupational hazards that can adversely affect cardiovascular health (43). Disparities in healthcare utilization further contribute to the sex difference, as men are less likely than women to seek preventive care, attend routine examinations, or adhere to medical advice, leading to delayed detection and management of key risk factors such as hypertension and hyperlipidemia (44). Collectively, these biological, behavioral, occupational, and healthcare-related differences likely explain the greater burden of CVD-DR observed in males.

Age-stratified analysis revealed higher ASDR and ASMR for CVD-DR among older age groups, consistent with the well-established role of aging as a major risk factor for CVD (45). Cardiac aging is characterized by reduced left ventricular contractility, diminished sympathetic nerve responsiveness, and lower ejection fraction, which decrease cardiac output and promote compensatory myocardial hypertrophy, ultimately leading to progressive functional decline (46, 47). Similarly, vascular aging involves arterial wall thickening, increased stiffness, and endothelial dysfunction, further impairing cardiovascular performance (48). In low- and low-middle SDI regions, the burden of CVD-DR was driven not only by driven not only by adults aged ≥60 years but also by individuals aged 25–59 years. In contrast, in high-middle and high SDI regions, the burden was predominantly concentrated among individuals aged ≥60 years. This shift in high-SDI regions is likely due to an aging population and better prevention strategies. These prevention strategies delay CVD onset in older patients. In low-SDI regions, limited healthcare resources, lower coverage of preventive measures, and persistent exposure to dietary and lifestyle risk factors in younger and middle-aged adults have led to a greater proportion of CVD-DR burden occurring before the age of 60 years. These findings highlight the need for age- and region-specific prevention and management strategies.

### 4.5 Burden analysis based on dietary risks

Globally, dietary risks contributing to CVD burden, ranked from highest to lowest, include diets low in fruits, high in sodium, low in whole grains, low in polyunsaturated fatty acids, low in vegetables, low in nuts and seeds, low in seafood omega-3 fatty acids, low in fiber, high in processed meat, low in legumes, high in trans fatty acids, high in red meat, and high in sugar-sweetened beverages. Among these, diets high in sodium and low in fruits and whole grains represented the primary dietary risks for CVD.

A high-sodium diet is the leading contributor to CVD-DR burden and its subtypes, including stroke-DR and AFF-DR. Sodium, primarily obtained from processed foods, ready-to-eat meals, and added salt during cooking, is essential in trace amounts but harmful in excess. However, Elevated sodium intake increases extracellular fluid volume, cardiac output, and vascular resistance, thereby raising the risk of hypertension, a major driver of CVD and a key determinant of stroke and AFF risk (49–51). Therefore, the WHO recommends a daily sodium intake of no more than 2.0 g (52), whereas the US Department of Health and Human Services recommends that specific populations, such as patients with hypertension and older adults, limit their intake to ≤1.5 g (53). Our analysis identified Central Europe and East Asia as hotspots for sodium-related CVD burden. Central Europe showed the highest percentage of deaths and the second-highest DALYs percentage, while East Asia had the highest DALYs percentage and the second-highest death percentage. These patterns are further reinforced by local dietary habits. Influenced by traditional diets rich in soy sauce and smoked foods, the average daily sodium intake in East Asia has significantly exceeded the WHO-recommended level (54). Although Japan has achieved notable salt reduction through decreases in sodium from pickled vegetables and miso paste, this progress has been offset by rising consumption of soy sauce, leaving sodium intake persistently high (55, 56). Similarly, in Central Europe, 73.4% of individuals from Poland consume excessive salt (57). Therefore, regions such as Central Europe and East Asia should implement more comprehensive and targeted salt reduction strategies, including nationwide public health campaigns to raise awareness about the harmful effects of high sodium intake, reformulation of processed and packaged foods to reduce sodium content, mandatory front-of-pack sodium labeling, promotion of low-sodium salt alternatives, and community-based dietary education programs. Such multilevel interventions are more likely to achieve sustained reductions in sodium intake and, consequently, mitigate the burden of CVD-DR.

A low-fruit diet was the second leading contributor to CVD-DR burden and the primary contributor to HHD-DR and AA-DR burden. Fruits are rich in potassium and various antioxidants, including polyphenols, flavonoids, and vitamin C, which reduce the risks of HHD and AA by exerting anti-inflammatory and antioxidant effects, improving vascular elasticity, and promoting renal sodium excretion (58). Consequently, the WHO recommends a daily intake of >400 g (59). South Asia was identified as the primary hotspot for fruit-deficient CVD burden, largely due to socioeconomic factors and regional dietary patterns. South Asia is one of the most densely populated regions globally, with nearly 400 people per square kilometer. Except for the Maldives and Sri Lanka, most South Asian countries are classified as low- or lower-middle-income. In such settings, limited household income can restrict fruit consumption, even when fruits are locally available (60). Jayawardena et al. (60) observed that fruit consumption in many South Asian countries remains below the WHO-recommended levels. In India, for example, fruit and vegetable consumption decreased steadily from 1973 to 2004, while meat and salt consumption increased (61). These findings suggest that economic constraints, rather than availability, limit the inclusion of fruit in the daily diet of low-income households. Notably, the widespread use of added salt and soy sauce also contributes to the prevalence of high-sodium diets in South Asia (62). Low fruit intake and high dietary sodium levels have a synergistic effect on elevating blood pressure, as potassium in fruits helps mitigate the hypertensive effects of sodium by promoting sodium excretion and improving vascular function (63). In populations with insufficient fruit consumption, this protective mechanism is weakened, thereby amplifying the blood pressure–raising effect of high-sodium diets (64). These findings underscore the importance of comprehensive dietary interventions in South Asia that simultaneously promote fruit consumption and reduce sodium intake to prevent and manage hypertension and related CVD.

A diet low in whole grains was the third most common contributor to CVD-DR burden and the primary driver of IHD-DR burden. Whole grains are rich in dietary fiber, potassium, and antioxidants, making them an important food source. Whole-grain diets reduce IHD risk by regulating lipid levels, blood glucose levels, and blood pressure (65, 66). The results of a meta-analysis indicated that for every 30 g/day increase in grain intake, the risk of CHD decreases by 6% (RR 0.94, 95% CI 0.92–0.97) (67). Therefore, the US Department of Agriculture and Department of Health and Human Services recommend a daily whole-grain intake of at least 48 g (68). In the present study, Central Asia was the primary hotspot for whole-grain-deficient CVD burden, likely reflecting dietary shifts accompanying economic development. For example, in Kazakhstan, with continuous economic growth, per capita meat consumption increased from 65.9 kg/year to 72.9 kg/year from 2011 to 2017, which may have displaced other foods, including whole grains (69). These findings suggest that public health strategies in Central Asia should emphasize increased whole-grain consumption as part of comprehensive CVD-DR prevention and management programs.

A diet high in processed meat is a major dietary risk factor for LEPAD-DR. Processed meats, which undergo salting, curing, smoking, fermentation, or chemical preservation, are rich in sodium chloride, nitrates, nitrites, phosphates, and saturated fats. The intake of processed meat is associated with a higher risk of peripheral artery disease (70), likely because its components exacerbate atherosclerosis and vascular damage (71, 72). In the present study, high-income North America was the primary hotspot for processed meat-related CVD burden, reflecting both economic levels and dietary patterns. With a robust economic foundation and an extensive industrial system, this region is a major producer and consumer of processed meats. Trade policies, such as the North American Free Trade Agreement, have further encouraged the production, trade, and consumption of agricultural products, including processed meats (73). Data indicate that processed meat intake in high-income North America significantly exceeds the global average, while a 30% reduction in intake could prevent an estimated 92,500 CVD cases nationwide (74, 75). These findings suggest that public health strategies in high-income North America should prioritize reducing processed meat consumption as part of comprehensive CVD-DR prevention and management programs.

Notably, dietary risks arising from insufficient intake of healthy foods include low consumption of fruits, whole grains, polyunsaturated fatty acids, vegetables, nuts and seeds, seafood omega-3 fatty acids, dietary fiber, and legumes. Addressing these deficiencies requires a multipronged approach. Public health authorities should strengthen nutritional education to raise awareness of the benefits of these foods, incorporate healthy eating guidance into school curricula, and promote dietary guidelines through mass media campaigns. Simultaneously, targeted subsidies and price incentives for healthy foods, particularly in low-income communities, can improve affordability and accessibility (76). Efforts to enhance food supply chains, establish farmers’ markets, and encourage local production can help ensure a stable supply of fresh, nutrient-rich foods year-round (76). Conversely, dietary risks from the excessive intake of unhealthy foods include high consumption of processed meat, trans fatty acids, red meat, and sugar-sweetened beverages. Effective prevention strategies should combine public education with fiscal measures, such as taxes on ultra-processed foods and sugar-sweetened beverages, legal limits on industrial trans fats, and clear front-of-package labeling to guide healthier choices (77). Supporting reformulation initiatives by the food industry to reduce sodium, added sugars, and saturated fats can further reduce population-level exposure to dietary risks.

### 4.6 Limitations and prospects

This study has several limitations. First, although the data cover 204 countries and territories globally, the completeness and accuracy of the information may vary across regions owing to differences in socioeconomic status, healthcare access, and reporting systems, particularly in low- and middle-income countries. Second, disease classifications and definitions in the GBD database may differ from international standards, potentially reducing the precision of burden estimates. Third, some data, including dietary and lifestyle information, were self-reported, which may have introduced misclassification bias. Fourth, the reliance on a single data source may lead to potential bias and may not capture all relevant influencing factors. Fifth, as an observational study, our analysis identified associations but could not establish causality. Furthermore, owing to limitations of the GBD database, we were unable to perform subgroup analyses for potential confounders such as physical activity, genetics, or other individual-level factors, which may influence the observed associations. Despite these limitations, this study provides a comprehensive overview of the global and regional burdens of CVD-DR. Future research integrating multiple data sources, incorporating standardized dietary and lifestyle information, and applying methods capable of inferring causality will help clarify the underlying mechanisms and inform prevention and management strategies.

## 5 Conclusion

From 1990 to 2021, the overall global CVD-DR burden increased, whereas the per capita burden decreased, primarily attributable to IHD-DR, stroke-DR, and HHD-DR. Regions with lower SDI face a disproportionately higher and more slowly declining CVD-DR burden. The primary dietary risks contributing to CVD burden were diets high in sodium, low in fruits, and low in whole grains. These findings highlight the need for region- and population-specific prevention and management strategies and underscore the critical role of a healthy diet in CVD management.

## Data Availability

The original contributions presented in this study are included in this article/Supplementary material, further inquiries can be directed to the corresponding author.

## Footnotes

1 ghdx.healthdata.org/gbd-2021/data-input-sources; accessed April 5, 2025
