# Supplementary material for: Global, regional, and national burden of cardiovascular disease due to dietary risks, 1990–2021
Source: Front Nutr. 2025 Oct 1;12:1623855. doi: 10.3389/fnut.2025.1623855 (PMC12520967; doi:10.3389/fnut.2025.1623855)
Supplement: Supplementary file 1 [file Table_1.docx]

**Supplementary Table S1** The definition of 13 specific diet risks associated with cardiovascular disease.

| Risk | Definition |
| --- | --- |
| diet high in trans fatty acids | Any intake greater than 0-1.1% daily energy of trans fat from all sources, mainly partially hydrogenated vegetable oils and ruminant products. |
| diet high in sugar-sweetened beverages | Any intake (in grams per day) of beverages with ≥50 kcal per 226.8 gram serving, including carbonated beverages, sodas, energy drinks, fruit drinks, but excluding 100% fruit and vegetable juices. |
| diet high in sodium | Average 24-hour urinary sodium excretion (in grams per day) greater than 1-5 grams. |
| diet high in red meat | Average of 0 grams per day (95% UI 0-200) of unprocessed red meat. Unprocessed red meat includes pork and bovine meats such as beef, lamb, and goat, but excludes all processed meats, poultry, fish, and eggs. |
| diet high in processed meat | Any intake (in grams per day) of meat preserved by smoking, curing, salting, or addition of chemical preservatives. |
| diet low in vegetables | Average daily consumption (in grams per day) of less than 280-320 grams of vegetables, including fresh, frozen, cooked, canned, or dried vegetables and excluding legumes and salted or pickled vegetables, juices, nuts and seeds, and starchy vegetables such as potatoes or corn. |
| diet low in fruits | Average daily consumption (in grams per day) of less than 310-340 grams of fruit including fresh, frozen, cooked, canned, or dried fruit, excluding fruit juices and salted or pickled fruits. |
| diet low in whole grains | Average daily consumption (in grams per day) of less than 140-160 grams of whole grains (bran,from breakfast cereals, bread, rice, pasta, biscuits, muffins, tortillas, germ, and endosperm in their natural proportion) pancakes, and other sources. |
| diet low in seafood omega-3 fatty acids | Average daily consumption (in milligrams per day) of less than 430-470 milligrams of eicosapentaenoic acid (EPA) and docosahexaenoic acid (DHA). |
| diet low in nuts and seeds | Average daily consumption (in grams per day) of less than 19-24 grams of nuts and seeds, including tree nuts and seeds and peanuts. |
| diet low in fiber | Average daily consumption (in grams per day) of less than 21-22 grams of fibre from all sources including fruits, vegetables, grains, legumes, and pulses. |
| diet low in legumes | Average daily consumption (in grams per day) of less than of 90-100 grams of legumes and pulses, including fresh, frozen, cooked, canned, or dried legumes. |
| diet low in polyunsaturated fatty acids | Average daily consumption (in % daily energy) of less than 7-9% total energy intake from polyunsaturated fatty acids. |
