# Supplementary figures and images for: Global, regional, and national burden of cardiovascular disease due to dietary risks, 1990–2021
Source: Front Nutr. 2025 Oct 1;12:1623855. doi: 10.3389/fnut.2025.1623855 (PMC12520967; doi:10.3389/fnut.2025.1623855)

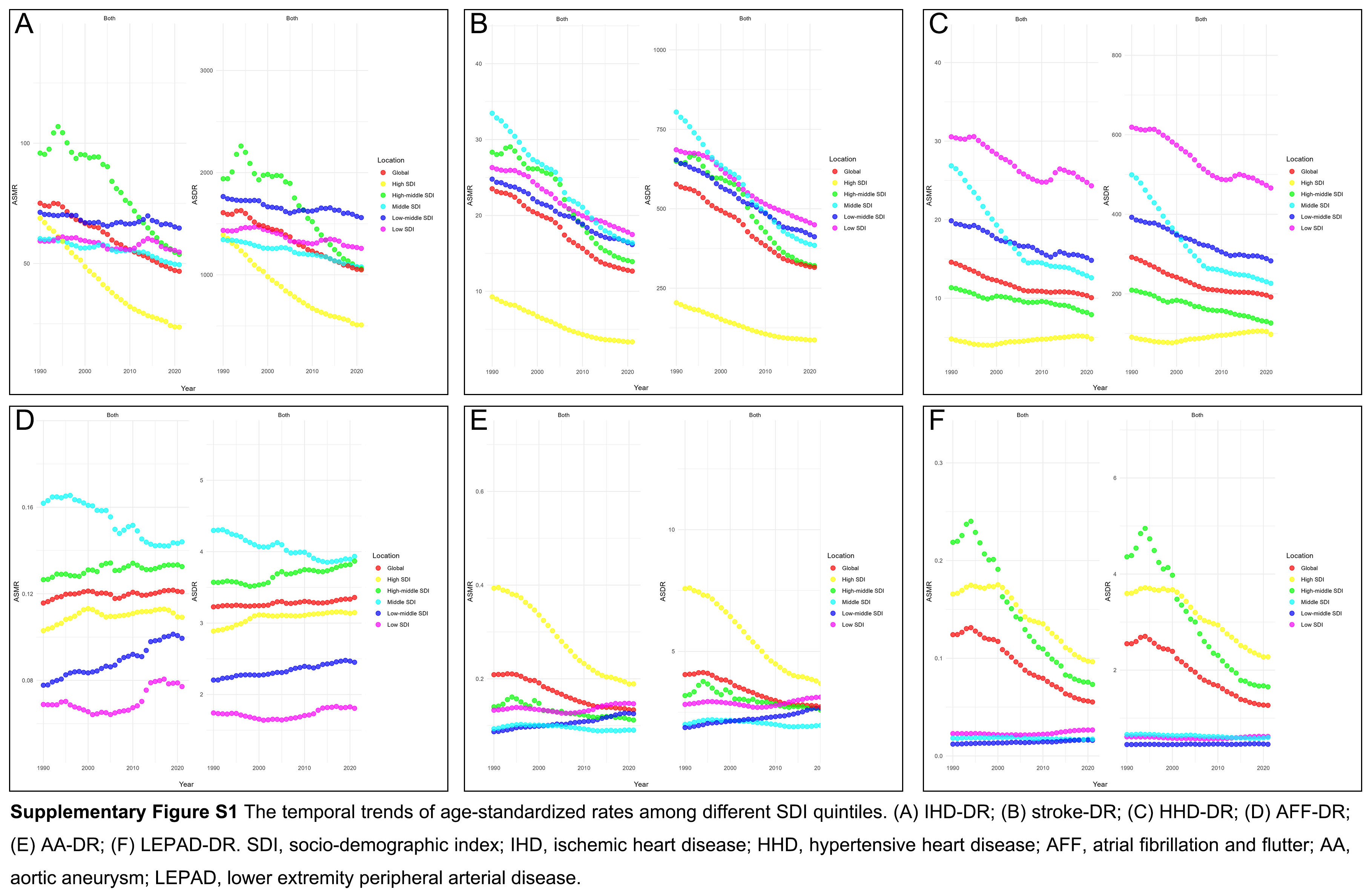

Supplement: Supplementary file 2 [file Data_Sheet_1.zip › Supplementary Figures/Supplementary Figure S1.jpg]

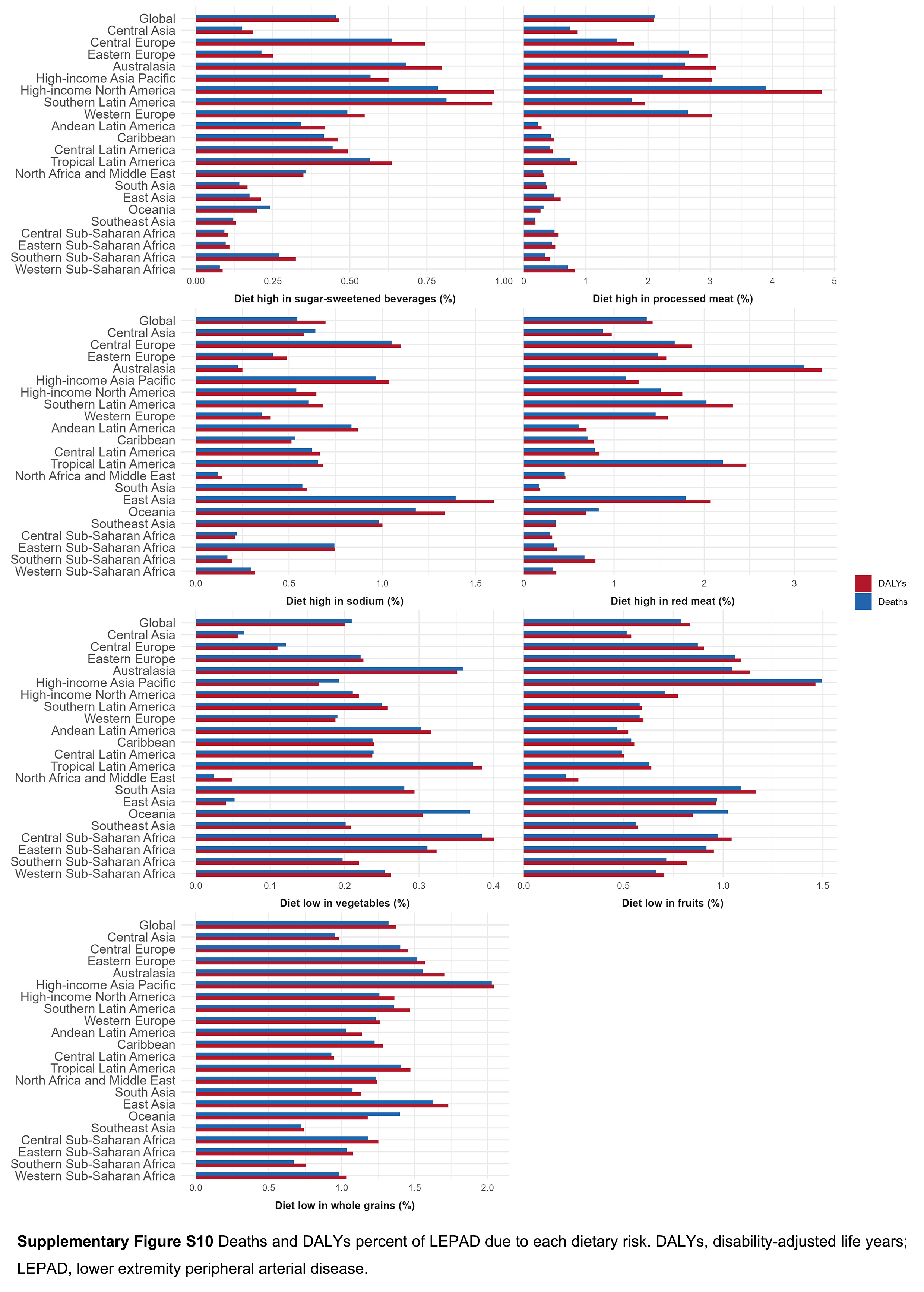

Supplement: Supplementary file 2 [file Data_Sheet_1.zip › Supplementary Figures/Supplementary Figure S10.jpg]

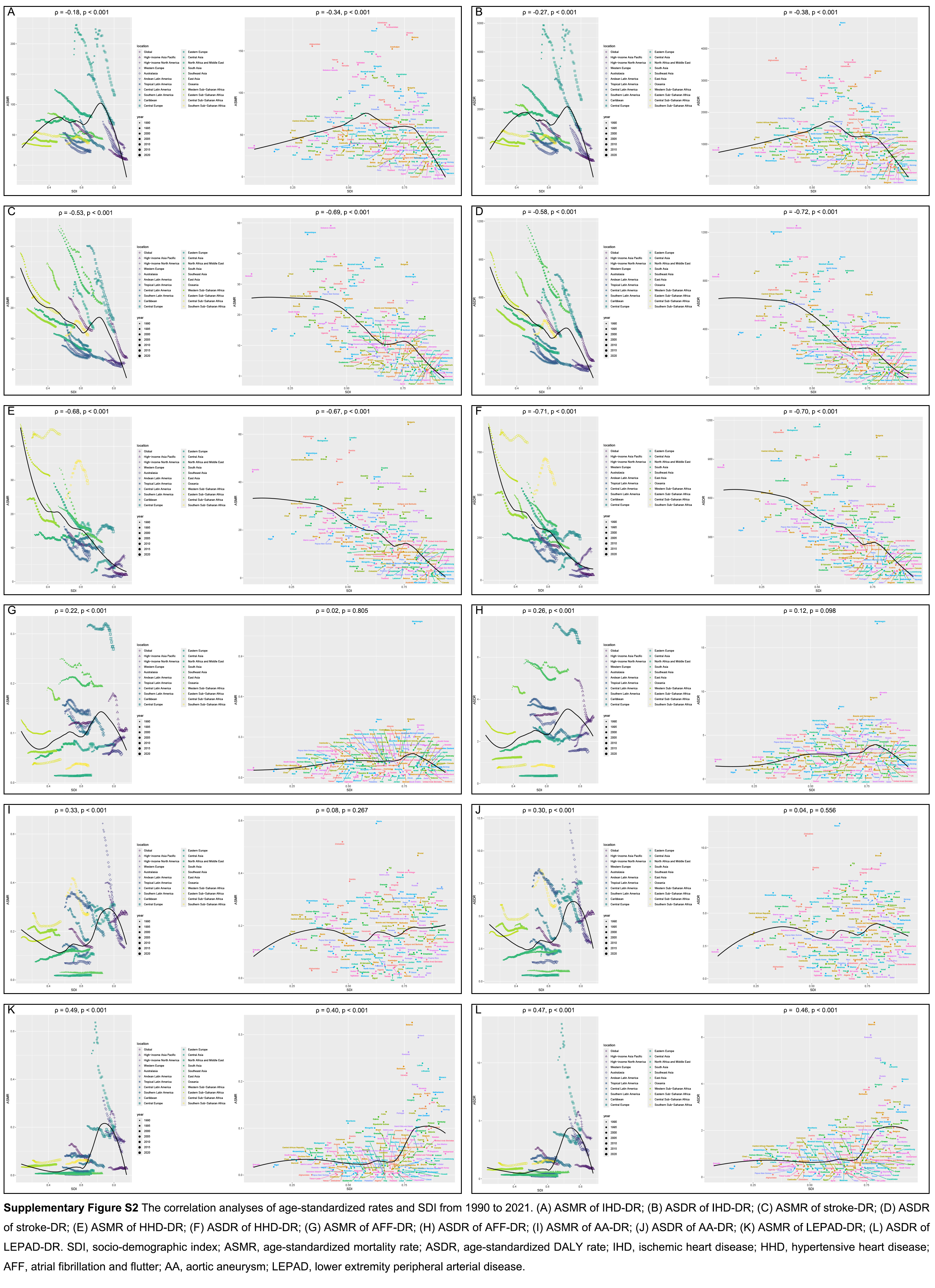

Supplement: Supplementary file 2 [file Data_Sheet_1.zip › Supplementary Figures/Supplementary Figure S2.jpg]

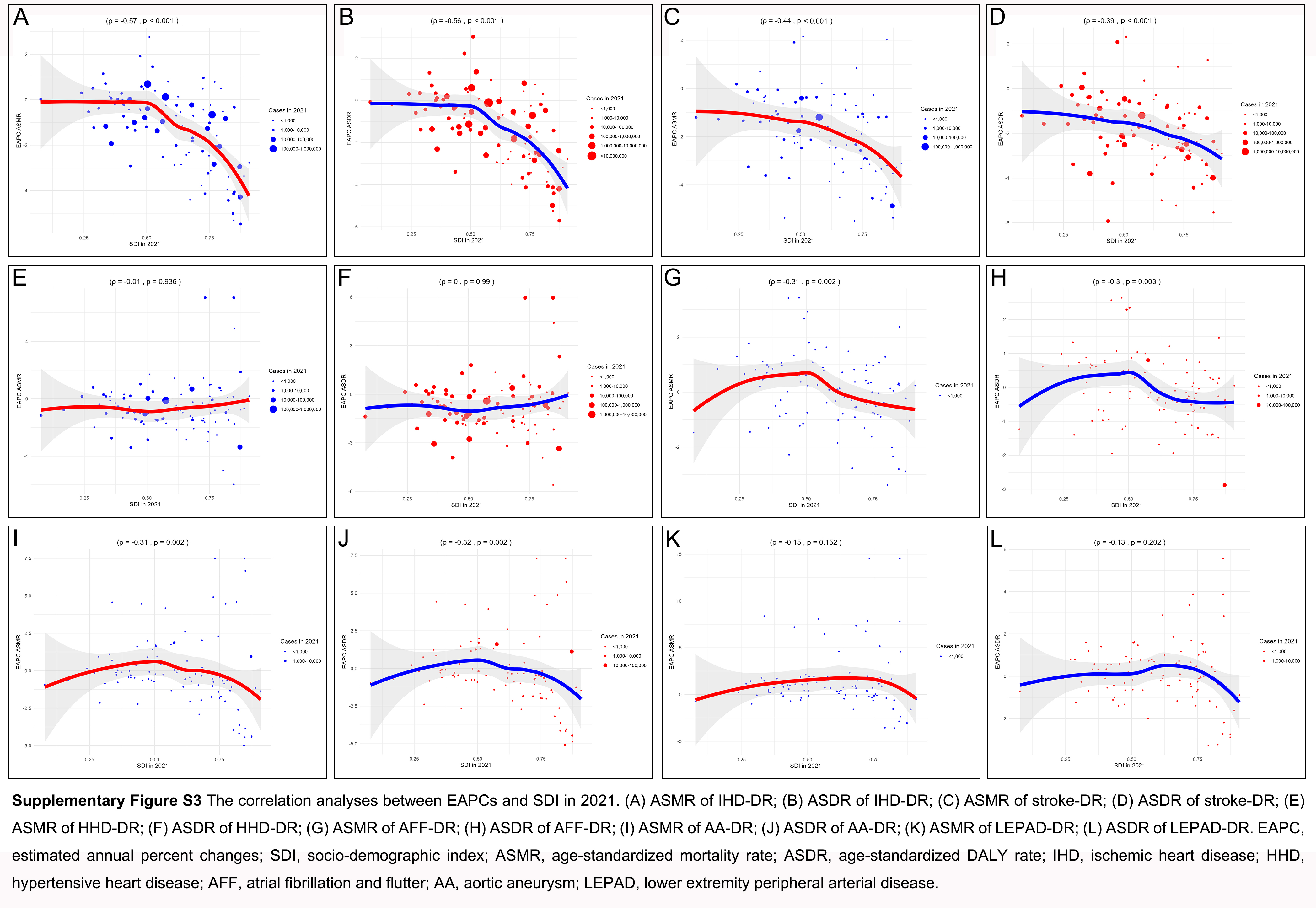

Supplement: Supplementary file 2 [file Data_Sheet_1.zip › Supplementary Figures/Supplementary Figure S3.jpg]

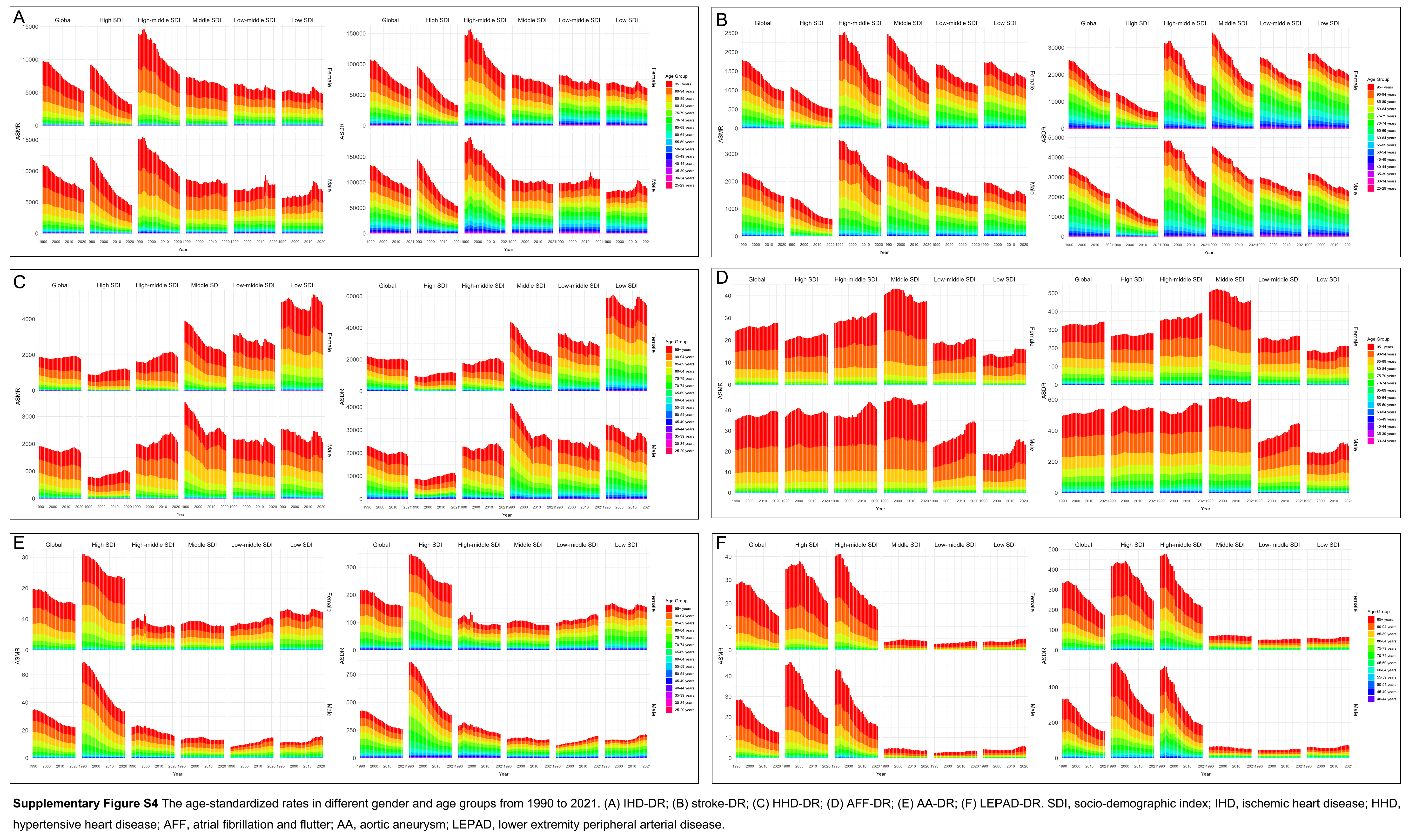

Supplement: Supplementary file 2 [file Data_Sheet_1.zip › Supplementary Figures/Supplementary Figure S4.jpg]

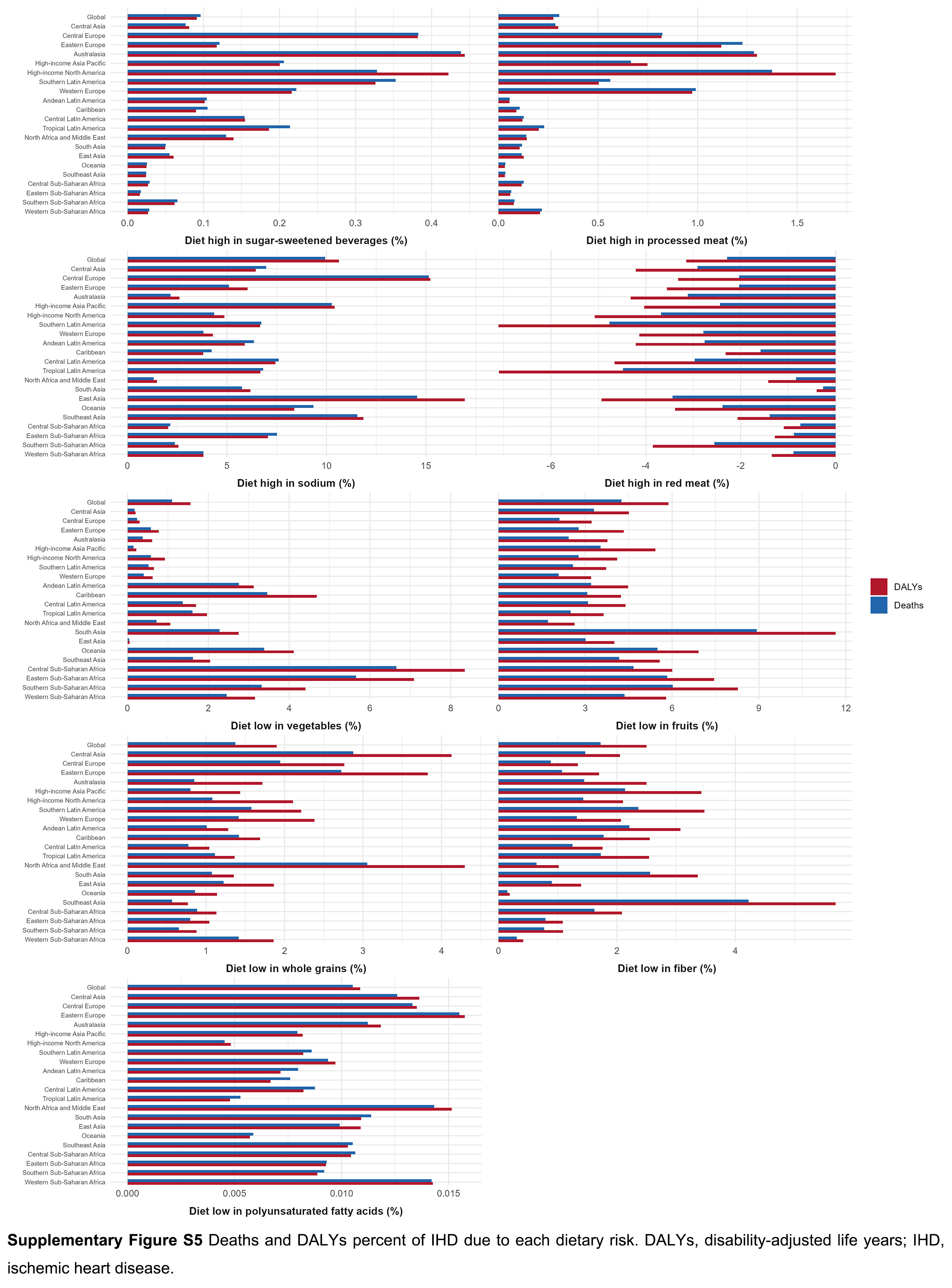

Supplement: Supplementary file 2 [file Data_Sheet_1.zip › Supplementary Figures/Supplementary Figure S5.jpg]

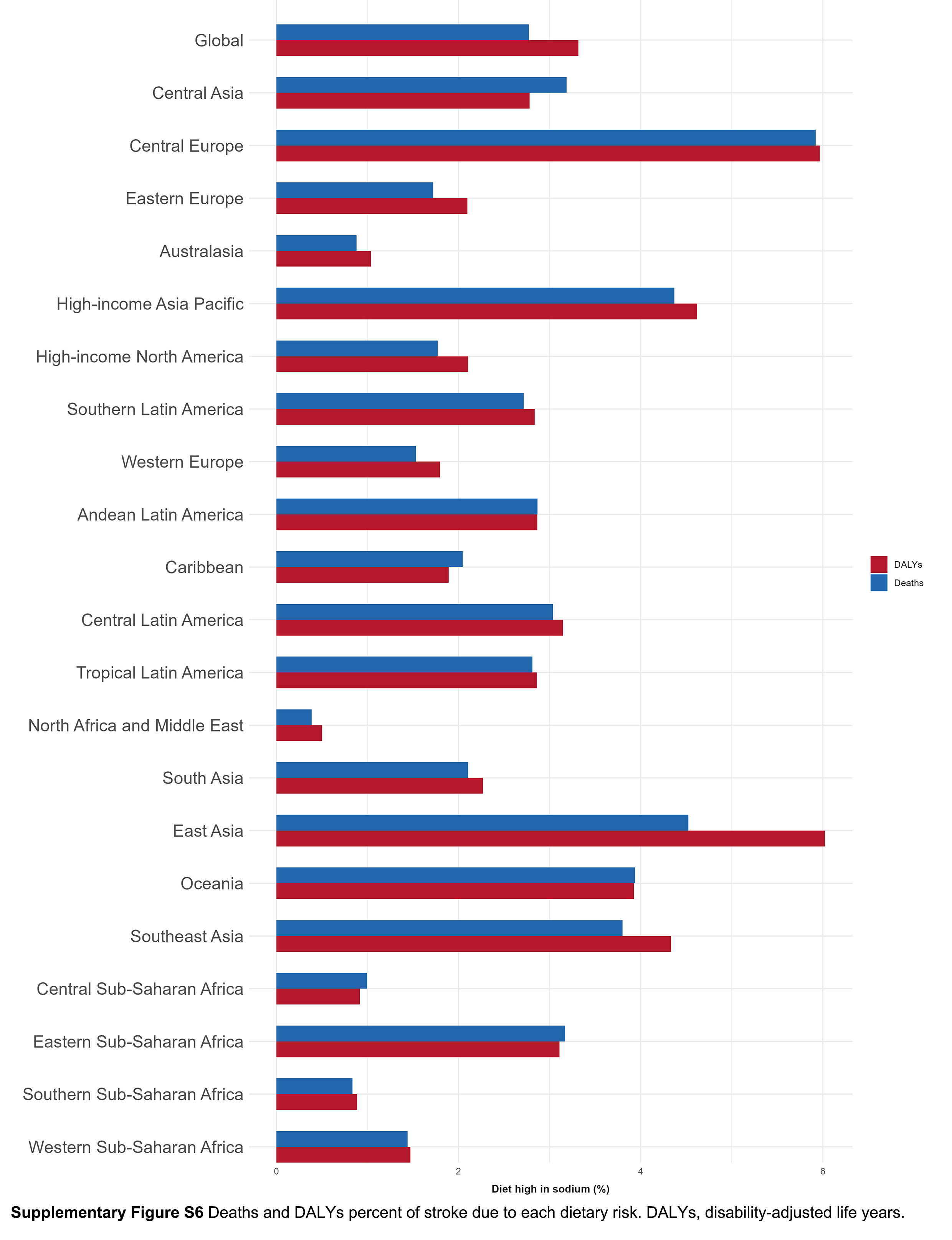

Supplement: Supplementary file 2 [file Data_Sheet_1.zip › Supplementary Figures/Supplementary Figure S6.jpg]

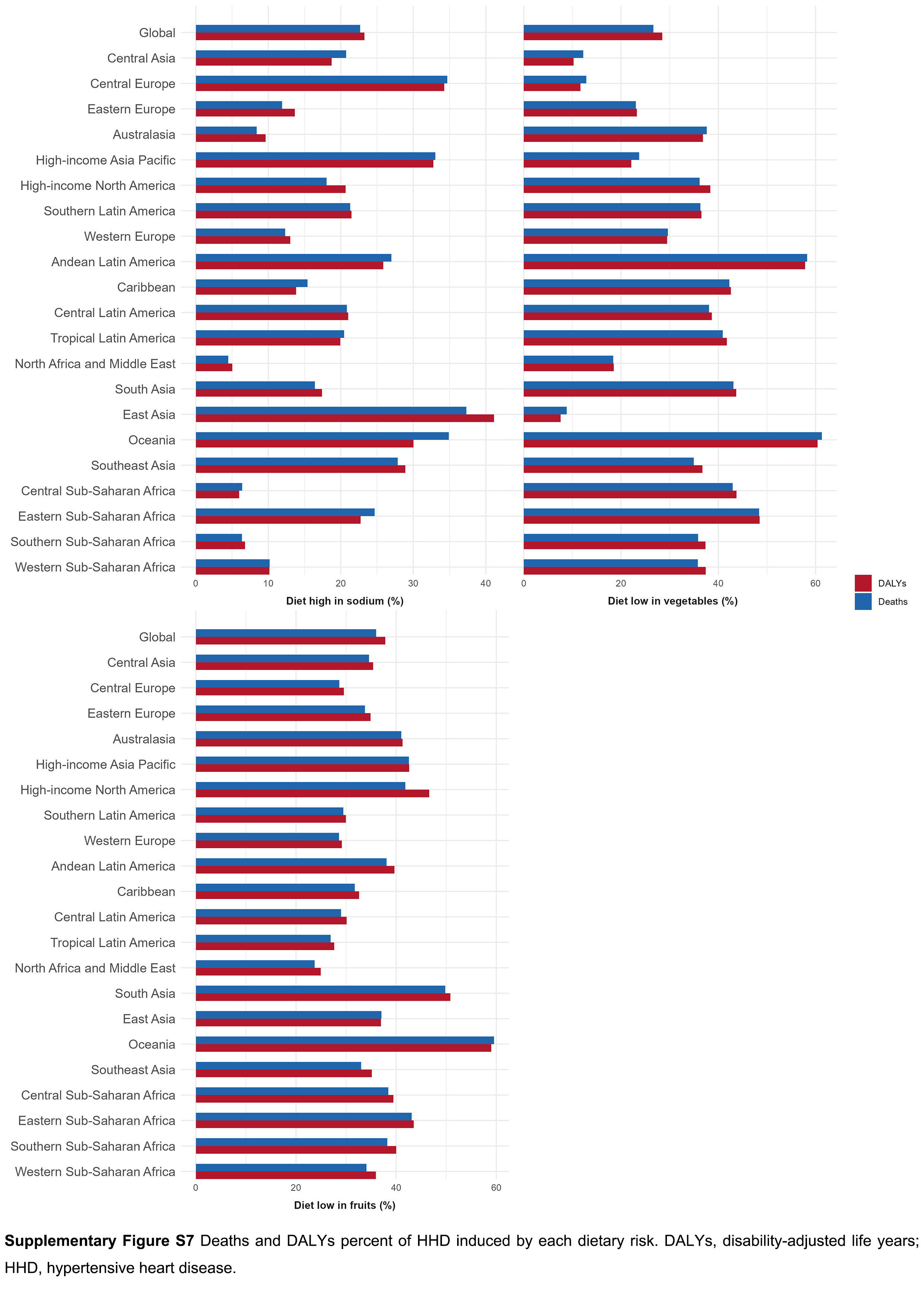

Supplement: Supplementary file 2 [file Data_Sheet_1.zip › Supplementary Figures/Supplementary Figure S7.jpg]

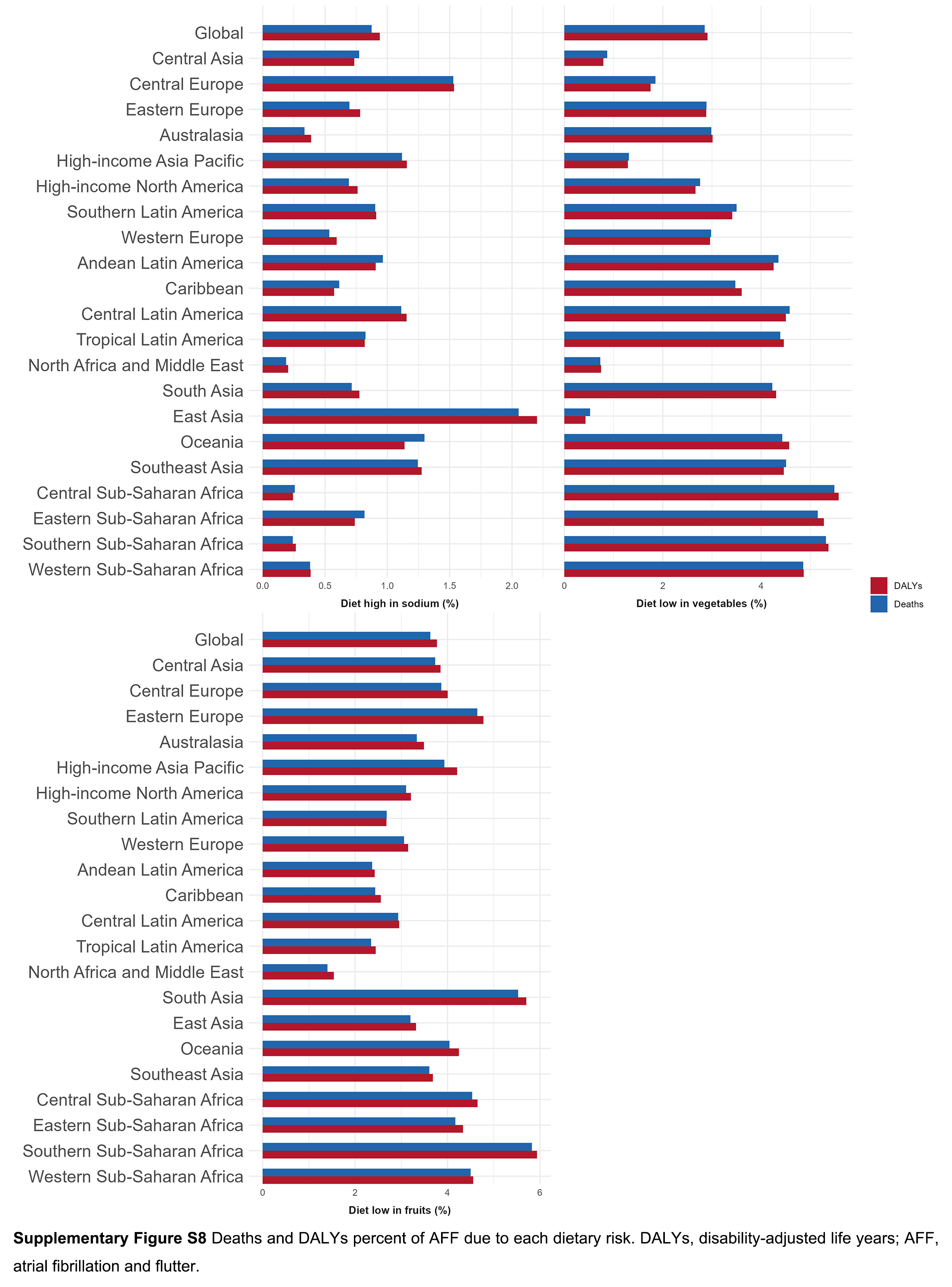

Supplement: Supplementary file 2 [file Data_Sheet_1.zip › Supplementary Figures/Supplementary Figure S8.jpg]

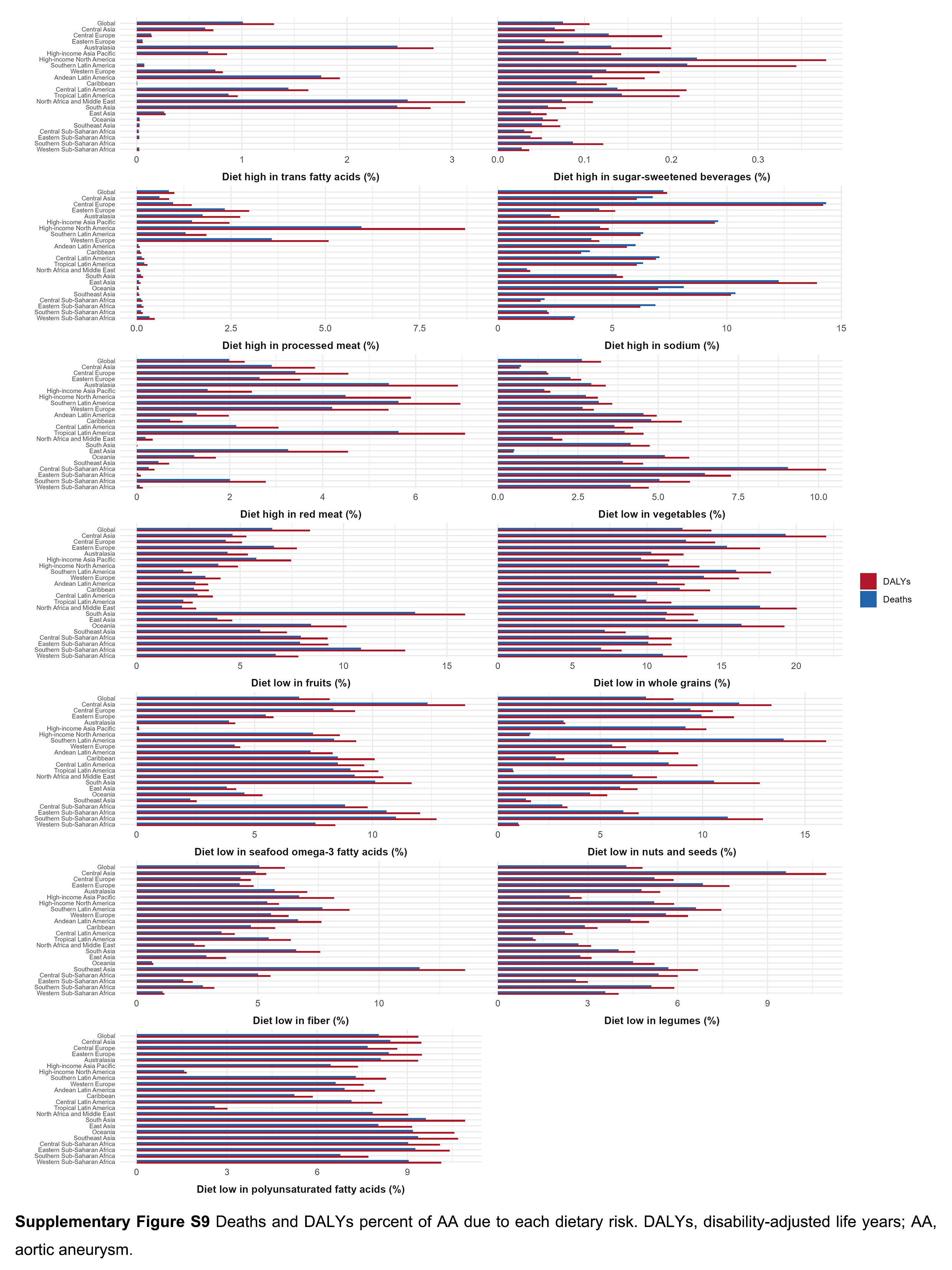

Supplement: Supplementary file 2 [file Data_Sheet_1.zip › Supplementary Figures/Supplementary Figure S9.jpg]
